# Serum lipid traits and the risk of dementia: A cohort study of 254,575 women and 214,891 men in the UK Biobank

**DOI:** 10.1016/j.eclinm.2022.101695

**Published:** 2022-10-06

**Authors:** Jessica Gong, Katie Harris, Sanne A.E. Peters, Mark Woodward

**Affiliations:** a The George Institute for Global Health, University of New South Wales, Level 5, 1 King St, Newtown, NSW 2042, Australia; b The George Institute for Global Health, Central Working - Fourth Floor, Translation and Innovation Hub, Imperial College London, 84 Wood Lane, London W12 0BZ, United Kingdom; c Julius Center for Health Sciences and Primary Care, University Medical Center Utrecht, Utrecht University, PO Box 85500, 3508 GA Utrecht, the Netherlands

**Keywords:** Dementia, Lipids, Apolipoprotein, UK Biobank

## Abstract

**Background:**

Serum lipid traits are associated with cardiovascular disease, but uncertainty remains regarding their associations with dementia.

**Methods:**

From 2006 to 2010, 254,575 women and 214,891 men were included from the UK Biobank. Cox regression estimated overall and sex-specific hazard ratios (HRs) for apolipoprotein A (ApoA), apolipoprotein B (ApoB), HDL, LDL, total cholesterol, triglycerides, lipoprotein A, and various lipid ratios, by quarters and standard deviation (SD) higher, associated with all-cause dementia, Alzheimer's disease (AD) and vascular dementia (VaD). Subgroup analyses by age and social deprivation were conducted.

**Findings:**

Over 11·8 years (median), 3734 all-cause dementia (1,716 women), 1231 AD and 929 VaD were recorded. Compared to respective lowest quarters, highest quarter of ApoA was associated with lower dementia risk (HR, [95% confidence interval (95% CI)]: 0·77 [0·69, 0·86]) while the highest quarter of ApoB was associated with greater risk (HR, 1·12 [1·01, 1·24]). Higher HDL/ApoA and ApoB/ApoA, were associated with greater risk of dementia (HR, 1·12 [1·00, 1·25], per standard deviation (SD), 1.23 [1·11, 1·37], per SD, respectively), LDL/ApoB was inversely associated (HR, 0·85 [0·76, 0·94], per SD. Higher triglycerides was associated with higher dementia risk in <60 years, but the inverse was observed for ≥60 years. Similar associations were observed for VaD and AD.

**Interpretation:**

Apolipoproteins, and their ratios, were associated with the risk of dementia. It may be prudent to consider apolipoproteins, along with circulating cholesterol, when assessing dementia risk.

**Funding:**

University of New South Wales, UK Medical Research Council, and the Australian National Health and Medical Research Council.

**Research in context**

**Evidence before this study**

While several factors involved in atherosclerosis have been identified as risk factors for dementia, the evidence remains uncertain for lipids. The associations between a wider panel of lipids, such as apolipoprotein A (ApoA) and apolipoprotein B (ApoB), as well as lipoprotein A (Lp(a)), with the risk of dementia have not been extensively examined. To date, evidence from large-scale population-based studies addressing demographic differences, including sex, age and socioeconomic status, were not available.

**Added value of this study**

To the best of our knowledge, this is the largest population-based study to examine a range of lipid traits and the risk of all-cause dementia and its main subtypes (Alzheimer's disease and vascular dementia), using data from the UK Biobank. Findings from the current study showed that apolipoproteins were associated with dementia risk. There was evidence for a difference by age group in triglycerides associated with dementia.

**Implications of all the available evidence**

Apolipoproteins, and their ratios, may be important markers for dementia. Routine examination of lipids panel with the inclusion of apolipoproteins should be considered and embedded in dementia prevention and risk reduction strategies.

Alt-text: Unlabelled box

## Introduction

Targeting potentially modifiable risk factors remains the best public health strategy for dementia on a population level, due to a paucity in effective disease-modifying treatments for dementia.^1^ Several well-established risk factors involved in atherosclerosis have also been identified for dementia, although the evidence remains inconclusive for lipids.^1^

A recent publication from the Clinical Practice Research Datalink (CPRD) cohort demonstrated a modest association between LDL cholesterol and dementia.^2^ While LDL cholesterol remains an important lipid fraction when evaluating general cardiovascular health, as well as brain health, other atherogenic lipoproteins, such as apolipoprotein A (ApoA) and apolipoprotein B (ApoB), were not evaluated in this large-scale study.^3^ Atherogenic lipoprotein particles are important proteins in the lipid metabolism that bind lipids (oil-soluble substances, such as fat and cholesterol) to form lipoproteins, and lipids can only enter the arterial wall within the apolipoprotein particle.^4^^,^^5^

Furthermore, the prevalence of dementia was estimated to be higher in women than men in 2019, with this preponderance expected to continue to 2050.^6^ Thus, there are strong imperatives to understand potential sex differences in biological mechanisms leading to dementia. In our previous study using the UK Biobank we showed that, whilst the rate of dementia was higher in men than women, several risk factors were associated with a greater risk of dementia in women than men,^7^ although we only examined circulating cholesterol in that study. A previous study in a Chinese population demonstrated that women are more susceptible to the adverse effects of serum LDL and total cholesterol on global cognition and mental status.^8^ Higher HDL cholesterol was associated with a greater cognitive decline in women but not in men.^8^

We thus assessed the comparative association of lipid traits with the risk of all-cause dementia as well as by dementia subtypes (vascular dementia and Alzheimer's disease) and evaluated potential effect modification by sex, in the large-scale UK Biobank. We also carried out analyses by age group and socioeconomic deprivation, as these are also key determinants of health, and effect modifiers for dementia risk.

## Methods

### Study population

From 2006 to 2010, more than 500,000 participants aged between 40 to 69 years were recruited into the UK Biobank. All participants attended one of the 22 assessment centres across the UK in England, Scotland, or Wales. Written informed consent was obtained for all participants. Consenting participants completed a touchscreen-based questionnaire, face-to-face verbal interviews, physical measurements, provided information on their medical history and medication use, and biological samples were also collected.

Follow-up for all participants involved linkage with hospital admissions data from England, Scotland, and Wales and the national death register to identify the date of the first known diagnosis of dementia after the date of baseline assessment. Death and hospital inpatient data were censored on the 30^th^ November 2020, or when death, fatal or non-fatal dementia, was recorded.

### Exposure measurements

The following lipid traits were measured in the blood sample collected at baseline recruitment: ApoA, ApoB, LDL cholesterol, HDL cholesterol, triglycerides, total cholesterol, and lipoprotein A (Lp(a)). Details on serum sample handling and assays in the UK Biobank have been described previously.^9^ Specifically, a series of biological samples were collected comprising 45 ml of blood and 9 ml of urine. Serum lipid traits were measured by immunoturbidimetric analysis on a Beckman automated haematology analyser (Beckman Coulter AU5800). Lipoprotein A was measured by immunoturbidimetric analysis using the Randox AU5800. Apolipoproteins were measured in g/L, circulating cholesterol and triglycerides were measured in mmol/L, and Lp(a) was measured in nmol/L. We also calculated the HDL/ApoA, LDL/ApoB, and ApoB/ApoA ratios, and all components of the ratios were converted to the same unit before dividing.

Correlation matrix for each lipid traits was generated, displaying Pearson's correlation coefficient between each lipid traits.

### Dementia outcome definitions

Incident fatal or non-fatal all-cause dementia diagnoses were identified using the International Classification of Diseases ICD-10 codes (A81.0, F00, F01, F02, F03, F05, G30, G31.0, G31.1, G31.8, and I67.3), (F00, G30) for Alzheimer's disease (AD) and (F01, I67.3) vascular dementia. If one or more of these codes were recorded as a primary or secondary diagnosis in hospital inpatient records from the Hospital Episode Statistics for England, Scottish Morbidity Record for Scotland and Patient Episode Database for Wales or recorded as the underlying or contributory cause of death in the death register data from NHS Digital in England and Wales and NHS Central Register, National Records of Scotland in Scotland. Outcome adjudication for incident dementia was conducted by the UK Biobank Outcome Adjudication Group.

### Covariates

Participants’ age, sex, and ethnicity were self-reported as a part of the touchscreen questionnaire at the study baseline. Blood pressure at baseline was taken as the mean of two sitting measures obtained from an Omron HEM-7015IT digital blood pressure monitor. Smoking status was self-reported and categorised as never, former, or current smokers. Self-reported diabetes was recorded (either type 1 or type 2 diabetes). Body mass index was calculated as the weight of the individual in kilograms, measured using the Tanita BC-418 MA body composition analyser, divided by the square of the individual's standing height in metres. Socioeconomic status was determined using the Townsend Deprivation Index, which is an area measure of social deprivation.^10^ Medication use was self-reported.

### Statistical analysis

For the current study, participants with prevalent dementia at the study baseline (n=263; 0·05%) were excluded, as were those without any measures of the lipid traits of interest (n=32,731; 6·5%). Baseline characteristics were presented as numbers (percentage) for categorical variables and as mean (standard deviation) for continuous variables by sex. Hazard ratios (HR) and 95% confidence intervals (CI) for dementia associated with the lipid traits were estimated using Cox proportional hazard models. Lipid traits were modelled as quarters (categorical), and continuously as sex-combined standard deviation higher. Quantitative confounders (Supplementary Figure 1) and their associations with dementia were assessed using the restricted cubic splines. Non-linear associations were observed for systolic blood pressure and body mass index; hence, they were included as equal fifths in the model adjustment, to account for the non-linearity. All analyses were adjusted for age, sex, ethnicity, smoking status, systolic blood pressure (in equal fifths), body mass index (in equal fifths), diabetes, Townsend deprivation index, lipid-lowering, and blood pressure lowering drugs.

We investigated whether the associations between lipids traits and dementia varied by sex, with the HR and 95% CIs calculated separately for women and men, and the interaction term between each risk factor and sex used to obtain the women-to-men ratio of hazard ratios (RHR) for each risk factor.^11^

Restricted cubic splines were constructed to explore non-linearity between exposures and the risk of dementia by sex, adjusted for the same set of covariates as outlined for the Cox regression models, using the “rcs” command in R, with 4 degrees of freedom, which equates to 3 internal knots at equally spaced percentiles.

Subgroup analyses were also conducted by age group (<60 years and ≥60 years) and by socioeconomic status, categorising individuals as above or below the national median Townsend score in the UK (-0·56), p values for interaction between the subgroups were reported. The effects of the lipid traits were also evaluated separately for Alzheimer's disease and vascular dementia using Cox regression models with the same adjustments used in models for all-cause dementia.

To determine whether each lipid trait, in addition to a model with traditional cardiovascular risk factors and LDL cholesterol, and a second approach of cardiovascular risk factors and total cholesterol, improved the prediction of incident dementia we used c-statistics and their difference. A model including each lipid trait and traditional cardiovascular risk factors, was compared to a (base) model including cardiovascular risk factors and LDL cholesterol, and traditional cardiovascular risk factors and total cholesterol. A higher c-statistic and positive difference of a model including lipid traits as compared with the base model with LDL or total cholesterol would demonstrate prognostic value of the lipid trait in predicting dementia above LDL or total cholesterol. All models were fit to the same complete case dataset that included 332,738 individuals to allow for direct comparison.

Additionally, as a sensitivity analysis, we also excluded people diagnosed with dementia or that died within the first five years, to minimize the effect of reverse causality.

Competing risk of mortality was assessed using the Fine and Gray proportional sub-distribution hazards regression models.^12^

All analyses were performed on complete case data using R Studio Version 4.1.0 (R Core Team, 2021).

### Ethics approval

The Research Tissue Bank approval from its governing Research Ethics Committee was obtained for the UK Biobank, as recommended by the National Research Ethics Service. The current research has been conducted using the UK Biobank Resource under the application No. 2495. Permission to use the UK Biobank Resource was approved by the access subcommittee of the UK Biobank Board. Written informed consent was obtained for all participants electronically.

### Role of the funding source

The funder of the study had no role in study design, data collection, data analysis, data interpretation, writing of the report or involved in the decision to submit the paper for publication.

## Results

### Baseline characteristics

Table 1 presents the baseline characteristics by sex among the 469,466 (54% women; mean age 56·5 years) participants with no prevalent dementia and with at least one lipid measurement at baseline were included in this study. The majority of the participants were of white ethnicity (94%). At the study baseline, a lower percentage of women were former or current smokers than men; and a lower percentage of women were using antihypertensive drugs or lipid lowering drugs. Women, on average, had a higher level of lipid traits than men, except for triglycerides. Details on baseline lipid traits by quarters, and the number of participants in each quarter were presented in Supplementary Table 1.

**Table 1:** Baseline characteristics after excluding those with baseline dementia and with all lipid traits missing at baseline in the UK Biobank, by sex.

Characteristics	Women (n=254 575)	Men (n=214 891)	Total (n=469 466)
Age (years) (Mean (SD))	56·3 (8·0)	56·7 (8·2)	56·5 (8·1)
Ethnicity:			
White	240 214 (94·4)	202 261 (94·1)	442 475 (94·3)
Other	13 345 (5·2)	11 441 (5·3)	24 786 (5·3)
Blood pressure (mmHg):			
Systolic (Mean (SD))	138·0 (20·0)	143·7 (18·8)	140·6 (19·8)
Diastolic (Mean (SD))	81·5 (10·8)	84·7 (10·8)	82·9 (10·9)
Smoking status:			
Never smoker	150 924 (59·3)	104 713 (48·7)	255 637 (54·5)
Former smoker	79 786 (31·3)	82 378 (38·3)	162 164 (34·5)
Current smoker	22 605 (8·9)	26 681 (12·4)	49 286 (10·5)
Diabetes:			
Type 1 diabetes	545 (2·1)	891 (4·1)	1 436 (3·1)
Type 2 diabetes	8 594 (3·4)	13 614 (6·3)	22 208 (4·7)
Body Mass Index (Mean (SD))	27·1 (5·2)	27·8 (4·2)	27·4 (4·8)
Social deprivation (national mean cut-off <-0·56):			
Higher SES	171 355 (67·3)	142 942 (66·5)	314 297 (66·9)
Lower SES	82 919 (32·6)	71 672 (33·4)	154 591 (32·9)
Drug use:			
Antihypertensive drugs	35 661 (14·0)	45 047 (21·0)	80 708 (17·2)
Lipid lowering drugs	27 410 (10·8)	42 976 (20·0)	70 386 (15·0)
Lipid traits:			
Apolipoproteins:			
Apolipoprotein A (Mean (SD)) (g/L)	1·63 (0·27)	1·43 (0·23)	1·54 (0·27)
Apolipoprotein B (Mean (SD)) (g/L)	1·04 (0·24)	1·03 (0·24)	1·03 (0·24)
Circulating cholesterol (mmol/L):			
HDL cholesterol (Mean (SD))	1·59 (0·38)	1·28 (0·31)	1·45 (0·38)
HDL cholesterol (Mean (SD)) (g/L)	0·62 (0·15)	0·49 (0·12)	0·56 (0·15)
LDL cholesterol (Mean (SD))	3·62 (0·87)	3·48 (0·86)	3·56 (0·87)
LDL cholesterol (Mean (SD)) (g/L)	1·40 (0·34)	1·34 (0·33)	1·38 (0·34)
Triglycerides (Median (Q1, Q3))	1·34 (0·97, 1·90)	1·69 (1·18, 2·45)	1·48 (1·05, 2·15)
Total cholesterol (Mean (SD))	5·87 (1·13)	5·48 (1·13)	5·69 (1·14)
Ratios:			
HDL/ApoA ratio	0·37 (0·04)	0·34 (0·04)	0·36 (0·04)
LDL/ApoB ratio	1·35 (0·09)	1·31 (0·10)	1·33 (0·10)
ApoB/ApoA ratio	0·65 (0·19)	0·74 (0·21)	0·69 (0·20)
Lipoprotein A (nmmol/L) (Median (Q1, Q3)):	22·2 (9·95, 62·0)	19·8 (9·17, 61·8)	21·1 (9·58, 61·9)

SD, standard deviation; SES, socioeconomic status; HDL, high density lipoprotein; LDL, low density lipoprotein.

Supplementary Figure 2 presented the correlation matrix displaying Pearson's correlation coefficient between each lipid traits.

### Lipid traits and all-cause dementia

Supplementary Figure 3 presented the directed acyclic graph, illustrating the potential relationships between lipids metabolism on dementia.

Over 11·8 years (median, interquartile range (IQR) = 8·3 to 9·7) follow-up, a total of 3734 all-cause dementia cases (1716 in women) were recorded. Of the 1716 women in this analysis with dementia, 271 were diagnosed at death only. Of the 2018 with dementia 343 were diagnosed at death only. Corresponding rates per 10,000 person years were 0·93 [0·79, 1·08] in women and 1·45 [1·24, 1·65] for men (Supplementary Table 2).

**Figure 1:**
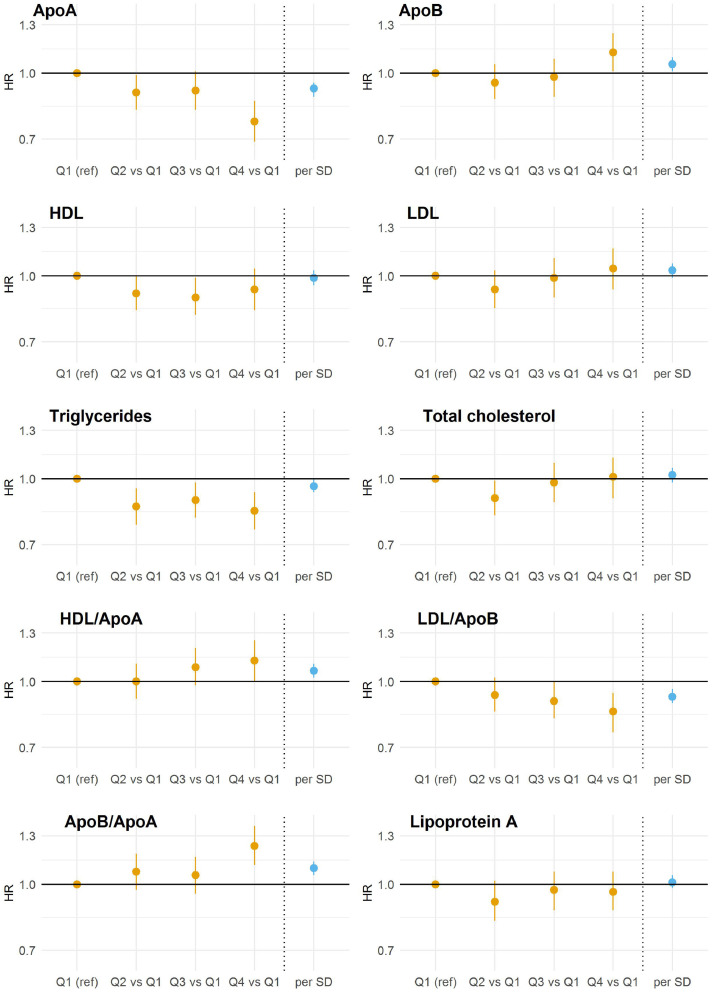
**Multiple-adjusted incident all-cause dementia hazard ratios for lipid traits by quarters and per standard deviation**. ApoA, apolipoprotein A; ApoB, Apolipoprotein B; HDL, high density lipoprotein; LDL, low density lipoprotein; SD, standard deviation; HR, hazard ratio. Adjusted for sex, age, ethnicity, Townsend Deprivation Index, body mass index, systolic blood pressure, smoking status, diabetes, lipids lowering drugs, blood pressure lowering drugs.

For ApoA, when compared with the lowest quarter (0·42 to 1·35 g/L), the highest quarter (1·7 to 2·5 g/L) had a lower risk of dementia (hazard ratio (HR) [95% confidence interval (95% CI)], 0·77 [0·69, 0·86]) (p for trend<0·001) (Figure 1; Supplementary Table 3); when modelled as a continuous measure, the HR [95% CI] per SD was 0·92 [0·88, 0·95].

The relationships between ApoB quarters and the risk of dementia were U-shaped, with the highest quarter (1·18 to 2·0 g/L) associated with a higher risk of dementia than the lowest quarter (0·4 to 0·86 g/L) (HR, 1·12 [1·01, 1·24]) (*p* for trend=0·043).

For HDL, LDL, and total cholesterol, when modelled by quarters, the associations with the risk of dementia appear to be U-shaped.

For triglycerides, when compared to the lowest quarter (0·23 to 1·05 mmol/L), the risk of dementia associated with the highest quarter (2·15 to 11·3 mmol/L) was lower (HR, 0·84 [0·76, 0·93]) (p for trend<0·003), and the HR per SD was 0·96 [0·93, 1·00].

For HDL/ApoA ratio, the highest quarter (0·39 to 1·41) was associated with a higher risk of dementia when compared with the lowest quarter (0·15 to 0·33) (HR, 1·12 [1·00, 1·25]) (p for trend=0.024). A higher HDL/ApoA ratio was associated with a greater risk of dementia (HR, 1·06 [1·02, 1·10], per SD).

For LDL/ApoB ratio, the highest quarter (1·39 to 2·72) was associated with a lower risk of dementia when compared with the lowest quarter (0·264 to 1·27) (HR, 0·85 [0·76, 0·94]) (p for trend=0.002). A higher LDL/ApoB ratio was associated with a lower risk of dementia (HR, 0·92 [0·89, 0·96], per SD).

For ApoB/ApoA ratio, when compared with the lowest quarter (1·69 to 5·45), the highest quarter (8·12 to 37·1) was associated with a greater risk of dementia (HR, 1·23 [1·11, 1·37]) (p for trend=0·001), and with a higher value indicated a greater risk of dementia (HR, 1·09 [1·05, 1·13], per SD).

For Lp(a), there was no evidence for a pattern of association with dementia risk: compared with the lowest quarter, the HR for the highest quarter was 0·96 [0·87, 1·07].

The association between each lipid trait and dementia were also presented per standardised unit higher (Supplementary Table 4).

### Subgroup analysis by sex, age, and social deprivation status

**Figure 2:**
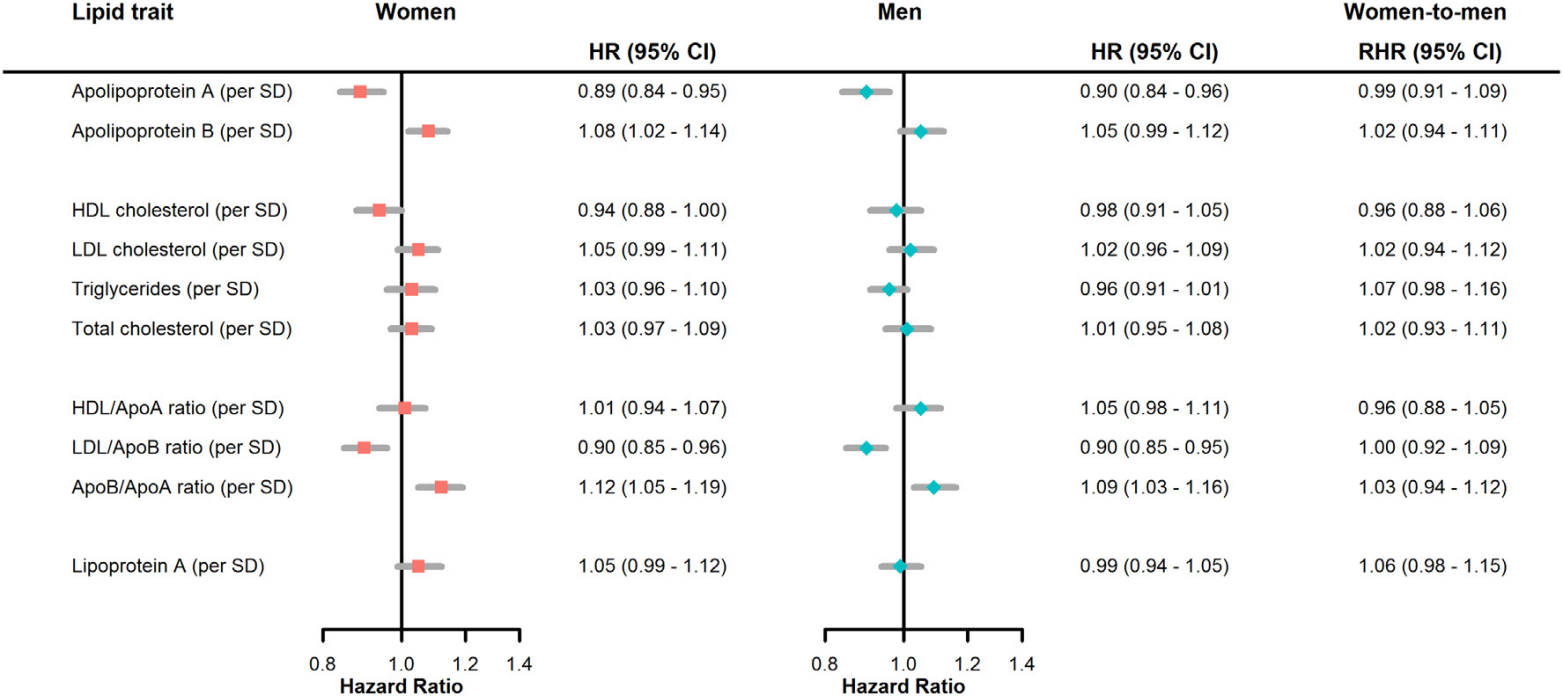
**Forest plot for multiple-adjusted incident all-cause dementia hazard ratios for lipid traits per standard deviation, by sex, and women-to-men ratio of hazard ratios**. ApoA, apolipoprotein A; ApoB, Apolipoprotein B; HDL, high density lipoprotein; LDL, low density lipoprotein; HR, hazard ratio; CI, confidence interval; RHR, ratio of hazard ratios; SD, standard deviation. Adjusted for age, ethnicity, Townsend Deprivation Index, body mass index, systolic blood pressure, smoking status, diabetes, lipids lowering drugs, blood pressure lowering drugs.

**Figure 3:**
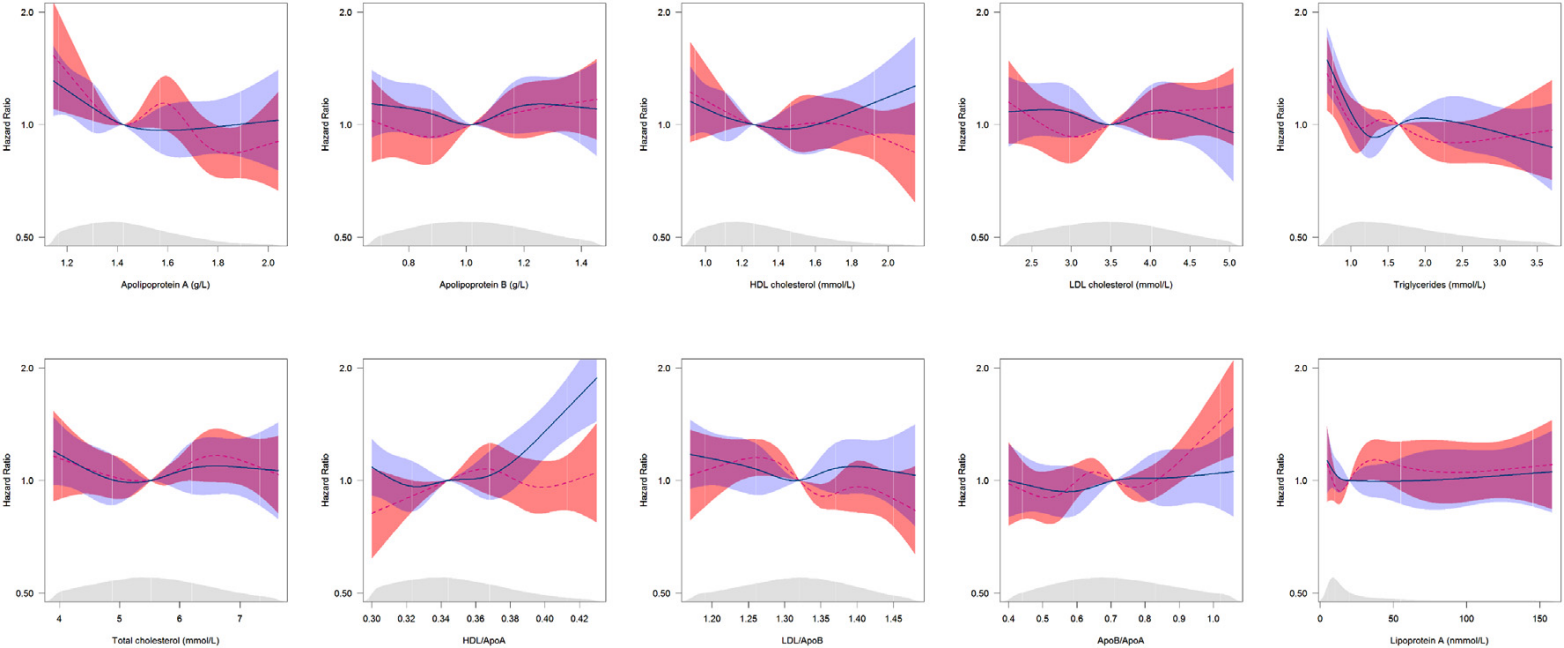
**Multiple-adjusted sex-specific restricted cubic splines (with kernel density plots) showing hazard ratios for the risk of incident all-cause dementia associated with lipid traits, by sex**. ApoA, apolipoprotein A; ApoB, Apolipoprotein B; HDL, high density lipoprotein; LDL, low density lipoprotein. Splines were adjusted for baseline age, ethnicity, smoking status, systolic blood pressure, body mass index, diabetes, Townsend index, lipids lowering drugs, blood pressure lowering drugs. Pink dotted line represents the hazard ratio for women, and the pink shaded area is the 95% confidence intervals for women; the solid blue line represents the hazard ratio for men, and the blue shaded area is the 95% confidence intervals for men. Extreme values in the upper and lower 5% of the lipid trait distribution were excluded. Reference values are the median value in women: Apolipoprotein A=1·42 g/L, Apolipoprotein B=1·02 g/L, HDL cholesterol=1·27 mmol/L, LDL cholesterol=3·49 mmol/L, Triglycerides=1·64 mmol/L, Total cholesterol=5·52 mmol/L, HDL/ApoA=0·34, LDL/ApoB=1·32, ApoB/ApoA=0·71, Lp(a)=19·91 nmmol/L.

There was limited evidence for a sex difference for lipid traits in association with the risk of dementia (Figure 2; Supplementary Figure 4; Supplementary Table 5). Sex-specific associations between lipid traits and dementia were also assessed using restricted cubic splines, indicating non-linearity in associations for all lipid traits; however, there was a clear separation in confidence intervals between women and men for higher values of HDL/ApoA displayed in the splines (Figure 3), with the association being J-shaped for men, but largely not associated with dementia risk, except at the lower end, for women.

When disaggregated by age group (<60 or ≥60 years), a higher level of triglycerides was associated with a lower risk of dementia in the ≥60 years age group, but with a higher risk of dementia in the <60 years age group (HR, 0·93 [0·89, 0·97] and 1·11 [1·02, 1·19] per SD, respectively; p for interaction<0·001) (Supplementary Figure 5; Supplementary Table 6). A higher HDL/ApoA ratio was associated with a higher risk of dementia in ≥60 years, but appeared to be U-shaped association in the <60 years age group (p for interaction=0·022).

The associations did not differ materially by socioeconomic status (Supplementary Figure 6; Supplementary Table 7).

### Lipid traits and dementia subtypes

**Figure 4:**
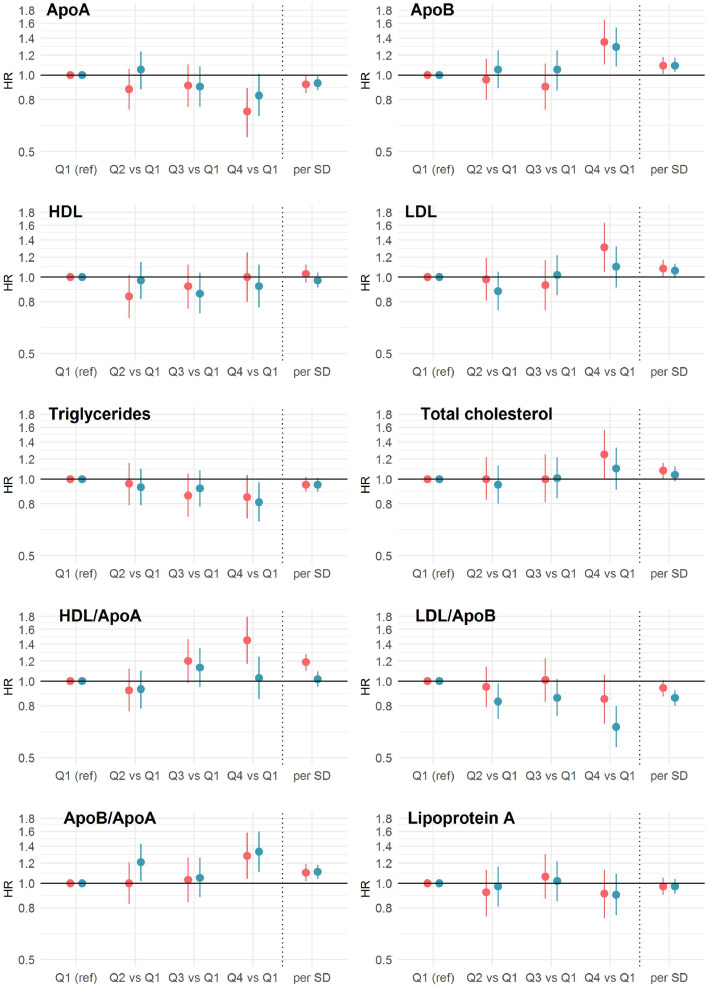
**Multiple-adjusted hazard ratios for lipid traits by quarters and per standard deviation, in associations with the risk of incident vascular dementia and Alzheimer's disease**. ApoA, apolipoprotein A; ApoB, Apolipoprotein B; HDL, high density lipoprotein; LDL, low density lipoprotein; SD, standard deviation; HR, hazard ratio. Adjusted for sex, age, ethnicity, Townsend Deprivation Index, body mass index, systolic blood pressure, smoking status, diabetes, lipids lowering drugs, blood pressure lowering drugs. Red represents vascular dementia, navy represents Alzheimer's disease.

A total of 929 vascular dementia (389 women) and 1,231 Alzheimer's disease (605 women) were recorded over the follow-up period. The associations between lipid traits and the dementia subtypes were broadly similar, with the exception of the HDL/ApoA ratio. When compared with the lowest quarter (0·152 to 0·33), the highest quarter (0·386 to 1·41) was associated with a higher risk of vascular dementia (HR, 1·37 [1·10, 1·70]), but of the same was not true for Alzheimer's disease (HR, 1·01 [0·83, 1·23]) (Figure 4; Supplementary Table 8).

### Sensitivity analyses

The inclusion of all lipid traits in addition to a base model of traditional cardiovascular risk factors and LDL cholesterol resulted in a higher c-statistic and positive differences for all lipid traits apart from total cholesterol (Supplementary Table 9), and all lipid traits compared with total cholesterol (Supplementary Table 10), the biggest differences compared with LDL cholesterol and total cholesterol were for models with ApoA, LDL/ApoB and ApoB/ApoA (per SD).

Excluding people diagnosed with dementia or who died within the first five years yielded similar hazard ratios to our primary analyses (Supplementary Table 11).

Findings allowing for the competing risk of mortality using Fine and Gray competing risk regression models were broadly similar to the main analyses from the Cox models (Supplementary Table 12).

## Discussion

Our study found associations between apolipoproteins and the risk of dementia: a higher level of ApoA was associated with a lower risk of dementia, and the association between ApoB and the risk of dementia was U-shaped. For circulating cholesterol, HDL, LDL, and total cholesterol there were U-shaped associations with dementia risk, and a higher level of triglycerides was associated with a lower risk of dementia. For the ratios, higher HDL/ApoA and ApoB/ApoA ratio were associated with a greater risk of dementia, while higher LDL/ApoB ratio was associated with a lower risk of dementia. The association appeared to be different for HDL/ApoA in women and men, and the associations were different for triglycerides and HDL/ApoA between <60 years and ≥60 years age groups. The associations were similar between socioeconomic status groups, and similar between vascular dementia and Alzheimer's disease.

A previous study using two Danish cohorts found that a higher triglycerides level was positively associated with the risk of non-Alzheimer dementia.^13^ While our findings from similar baseline age structure (<60 years) was largely consistent with the Danish study, there was a marked difference between triglycerides measured in mid-life (<60 years) versus late-life (≥60 years) in relation to dementia risk, with those with triglycerides measured in late-life at study baseline showed a lower dementia risk, indicating possible different mechanisms and effects in contributing to dementia risk across the lifespan.

Recent studies showed that apolipoproteins might be more strongly associated with cardiovascular events than circulating cholesterol alone.^4^^,^^14^ A Mendelian Randomisation (MR) study showed that ApoB is the predominant trait in the relationship of lipoprotein lipids with the risks of heart disease and stroke.^15^ In a separate MR study, ApoB increased the risk of diabetes after accounting for LDL cholesterol.^16^

The underlying mechanism between lipid traits and dementia risk remains unclear,^2^^,^^17^ which has been linked to adverse vascular outcomes such as atherosclerosis and cerebrovascular conditions,^17^ and may also contribute to Alzheimer's neuropathology.^2^ Importantly, the ApoE gene is associated with Alzheimer's disease, and is involved in cholesterol transportation in the brain, and the lipids in the brain may then influence the function of the β- and γ-secretase, which are cleavage enzymes involved in Aβ formation and aggregation.^2^ However, it is uncertain whether circulating cholesterol reflects the lipid concentration in the brain, since the blood-brain barrier prevents the transportation of lipids directly from plasma to the central nervous system.^2^

Previous studies analysing associations between apolipoproteins and the risk of dementia have provided conflicting results. In a cohort after 15-year follow-up, higher level or atherogenic lipoproteins including ApoB and LDL cholesterol were associated with greater cognitive decline in executive function.^18^ In the Sydney Memory and Aging Study, a lower ApoA1 level and higher ApoB/ApoA1 ratio were associated with a greater risk of cognitive decline.^19^ In the Honolulu-Asia Aging Study cohort of Japanese-American men, those in the highest quarter of ApoA1 had a significantly lower risk of dementia than those in the lowest quarter.^20^ An MR study in the UK Biobank with participants reporting on disease outcomes for their first-degree relatives found that, while there was a genetic liability for ApoB associated with a greater risk of Alzheimer's disease and dementia, the associations attenuated after taking ApoE genotype into account.^16^ Despite the MR study design, the authors indicated several caveats associated with the study, including that the genetically elevated ApoB is only an approximation to unconfounded estimates within participant's first-degree relatives, subject to reporting bias and measurement error, and causality cannot be inferred.^16^

ApoB is the main structural component of atherogenic lipoproteins and can be a direct measure of the total circulating atherogenic particles, as they can then enter the arterial wall while containing the mass of cholesterol (usually LDL cholesterol) within them.^5^ The ‘response-to-retention’ hypothesis^21^ detailing the entrapment of ApoB containing particle within the tunica intima arterial wall as a necessary step in order for the process of atherosclerosis to initiate.^5^^,^^15^^,^^21^ From experimental animal models, mice that were overexpressing ApoB exhibited hyperlipidaemia, neurodegeneration, amyloid plaques accumulation, and cognitive impairment, similar to transgenic mouse model overexpressing proteins related to Alzheimer's disease.^22^^,^^23^ Our study provided further empirical evidence in line with these pathophysiological hypotheses for apolipoproteins, even in the absence of any, or very weak associations between circulating cholesterols and dementia, non-linear relationship between ApoB and dementia was observed. While it may be due to reverse causation, after excluding the people diagnosed with dementia or that died within the first five years, the U-shaped association remained robust for ApoB and dementia.

Notwithstanding the growing evidence for lipids being potential predictors for dementia,^2^^,^^24^ dyslipidaemia was not included as one of the twelve potentially modifiable risk factors for dementia outlined in the Lancet Commission Report.^1^ Intervention studies involving lipid-lowering treatments, such as statins, which is considered as the first-line treatment for lowering ApoB level, have not yielded sufficient evidence from clinical trials in favour of its therapeutic benefits in preventing cognitive decline or dementia.^25^ Albeit observational studies demonstrating lower risk of dementia in association with statin use, conflicting results remain around the benefits of statin on reducing the risk of dementia caused by cerebrovascular diseases.^26^ The assessment of a comprehensive panel of lipid traits (beyond cholesterols) should be considered to identify at-risk individuals. ApoB from cerebrospinal fluid sample was identified as an important marker for tau pathology and cognitive decline,^27^ which reinforces the need for considering apolipoproteins in clinical practice, and these findings may also be useful for designing more effective drug interventions.

Proatherogenic and antiatherogenic lipoprotein measurements, and their ratios, may capture important clinical risk information on dementia risk variations, as has been highlighted for CVD outcomes previously.^14^^,^^28^^,^^29^ It is possible that the level of circulating cholesterol without commensurate apolipoproteins indicates different pathophysiological changes, which may be involved in dementia physiopathology. For instance, discordant LDL/ApoB may indicate a decreased LDL clearance due to impaired LDL receptors, has been associated with insulin resistance, increased systemic inflammation and lower serum lipids in circulation.^30^ Conversely, where there is a comparatively higher level of ApoB concentration than circulating LDL cholesterol, the increased uptake of ApoB particles by the pancreas could then mean that there is a reduction in the function of beta cells.^16^ Without discounting the importance of circulating cholesterols on cardiovascular and dementia risk, our study instead highlights the need to consider apolipoproteins in tandem with circulating cholesterol level, to provide a more accurate estimation of lipid profile and the risk of dementia.

Furthermore, higher HDL/ApoA was associated with a greater risk of dementia. The potential mechanism was previously illustrated for cardiovascular risk, such that a greater HDL/ApoA ratio predicts increased risk of atherosclerosis.^14^ Vascular contributions to cognitive impairment and dementia (VCID) is well recognised, with atherosclerosis playing a crucial role in dementia aetiology alongside neurodegeneration.^31^ Notwithstanding the inconsistent evidence around HDL cholesterol levels in association with dementia risk,^2^^,^^32^^,^^33^ ApoA, and its ratio with HDL cholesterol, may be important indicators to be included in risk assessment, with previous experimental studies suggesting ApoA is also involved in amyloid pathology and astrocyte reactivity.^34^ Lastly, ApoB/ApoA ratio has been shown to be a strong predictor for cardiovascular risk^4^; and given the atherogenic effects of these apolipoproteins, a higher ApoB/ApoA ratio which may indicate an overall greater atherogenic effect which was associated with greater risk of dementia, consistent with cardiovascular risk reported in previous studies.^4^^,^^29^^,^^35^

Sex differences in lipid metabolism have previously been highlighted, and sex hormones were thought to play an important role in regulating plasma lipid kinetics.^36^ In comparison to men, pre-menopausal women have less proatherogenic plasma lipid profile^36^; while male sex, aging in women, and menopause have been associated with increased level of ApoB concentration,^37^ with the effects of physiological changes in hormonal milieu, and potentially exogenous hormone use may have effects on plasma lipid kinetics and their concentration level.^36^ Sex differences exist in plasma lipid and lipoprotein particle concentration, some differences in subclass distributions and sizes were also noted, which may account, at least in part, for the sex differences in cardiovascular risk profile.^36^ While biological differences may be plausible in explaining the sex differences in lipid traits and dementia risk, sometimes treatment disparities, reflecting potential suboptimal treatments, may be responsible for differences in disease risk between men and women. Future studies should continue to disaggregate analyses by sex to uncover any potential sex differences in lipid traits associated with dementia risk.

Participants in the UK Biobank are predominantly white and relatively affluent individuals, with a low response rate to its baseline survey. However, with its large sample size and variety of exposures, risk factor associations in UK Biobank seem to be generalizable, based on a comparison with data from 18 prospective cohort studies (Health Surveys for England and the Scottish Health Surveys).^38^^,^^39^ However, valid assessment of exposure-disease relationships may be widely generalizable and does not require participants to be representative of the population at large.^40^ Second, while it is important to examine dementia by subtypes, there are a limited number of cases with Alzheimer's disease and vascular dementia in the UK Biobank, limiting statistical power to detect true associations. Third, we did not have information on the types of cholesterol-lowering treatments reported by the participants. Fourth, we do not have information on the Apolipoprotein E (ApoE) genetic variants, with ApoE being the main apolipoprotein expressed in the brain and a known genetic variant that is strongly associated with Alzheimer's Disease. Fifth, the use ICD codes to define dementia using hospital admission and mortality data may be subject to misdiagnosis or misclassification. This definition does not sufficiently capture those living with dementia in the community who has never been hospitalised, which may be subjected to gender bias, given the possible differences in multimorbidity^41^ and frailty between women and men,^42^ which may lead to subsequent hospitalisations. Sixth, false positives can be expected due to multiple testing, and hence borderline results need to be interpreted with caution. Seventh, the built-in selection bias of hazard ratios should be mentioned, such that the hazard ratio estimated in this study may be period-specific.^43^ Given that hazard ratios may change over time, this study does not consider the time-varying effect of the exposure and confounders, and the estimates from the study depend on the length of follow-up and may differ when a longer follow-up is considered.^44^ Lastly, all observational studies, even those with large sample sizes, are in jeopardy from reverse causality and residual confounding, and hence not suitable for making causal inferences. However, those with dementia at baseline were excluded from the analyses, and in the sensitivity analyses we excluded people diagnosed with dementia or died within the first five years, to reduce the effect of reverse causation. Future studies applying a causal inference framework (i.e., Mendelian Randomisation and randomised controlled trials) are needed to confirm the direction of the relationships. Findings need to be replicated in future studies, ideally with longer follow-up time and more dementia events.

Apolipoproteins, and their ratios, may be important markers for dementia and thus may be appropriate targets for lipid-modifying therapeutics. Routine examination of lipids panel with the inclusion of apolipoproteins, alongside with other lipid traits, and lipids management should be considered and embedded in the dementia prevention and risk reduction strategies.

## Contributors

JG conceptualized the study, conducted the formal analyses, visualization, and wrote the original drafts of the manuscript. MW and SAEP provided input to modify the study design. MW, SAEP and KH provided statistical support, supervision, and reviewed the manuscript. JG and KH had full access and verified all the data in the study. All authors were involved in interpretation of results and accept responsibility to submit for publication.

## Data sharing statement

The data that support the findings of this study are available from the UK Biobank but restrictions apply to the availability of these data, which were used under license for the current study, and so are not publicly available. The UK Biobank resources are however available from the authors upon reasonable request and can be accessed through applications on their website (https://www.ukbiobank.ac.uk/).

## Declaration of interests

MW does consultancy for Amgen and Freeline outside the submitted work; no support from any organisation for the submitted work; no other relationships or activities that could appear to have influenced the submitted work. All other authors have nothing to declare.

## Appendix Supplementary materials

Image, application 1
