# Supplementary material for: Serum lipid traits and the risk of dementia: A cohort study of 254,575 women and 214,891 men in the UK Biobank
Source: eClinicalMedicine. 2022 Oct 6;54:101695. doi: 10.1016/j.eclinm.2022.101695 (PMC9561731; doi:10.1016/j.eclinm.2022.101695)
Supplement: Supplementary file 1 [file mmc1.docx]

**Supplementary Material**

**Supplementary Figure 1: Multiple-adjusted sex-specific restricted cubic splines (with kernel density plots) showing hazard ratios for the risk of incident all-cause dementia associated with various quantitative confounders**

| 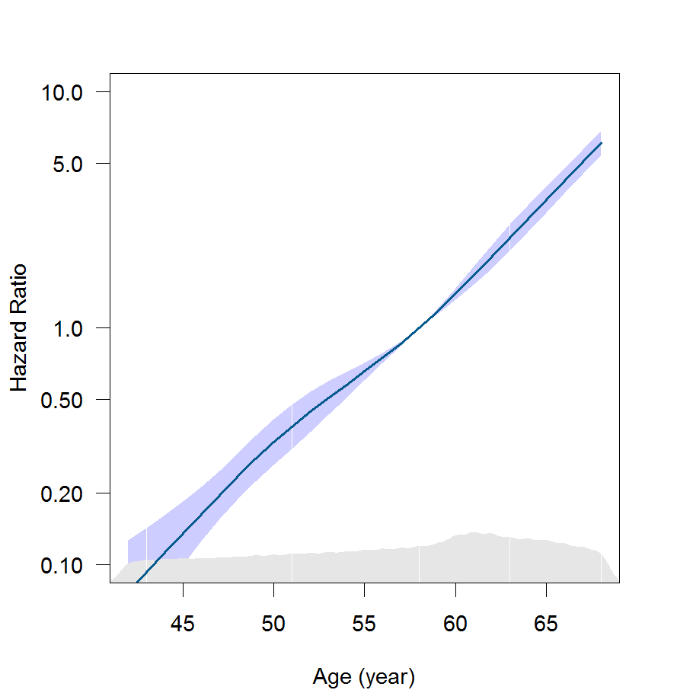 | 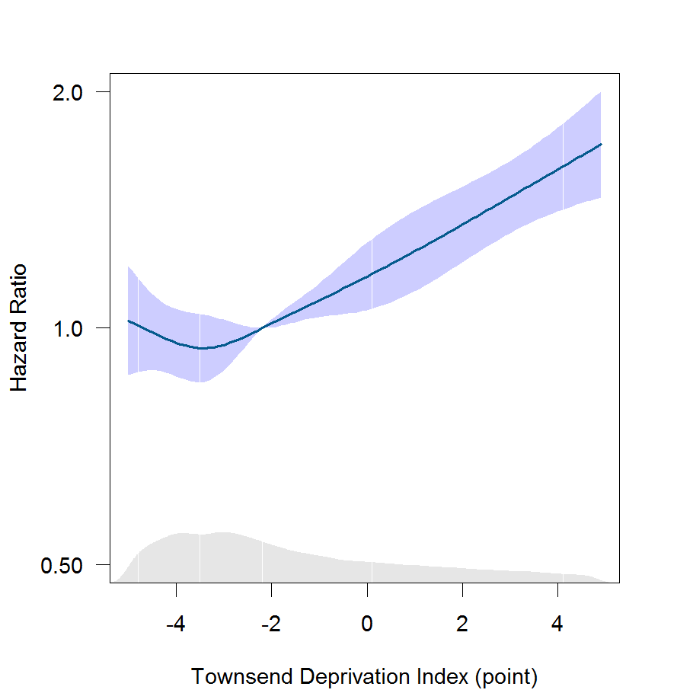 |
| --- | --- |
| 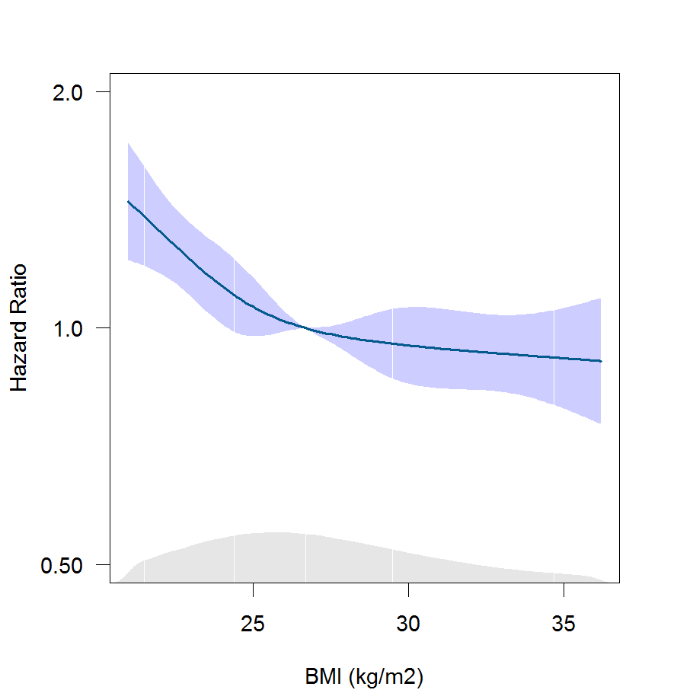 | 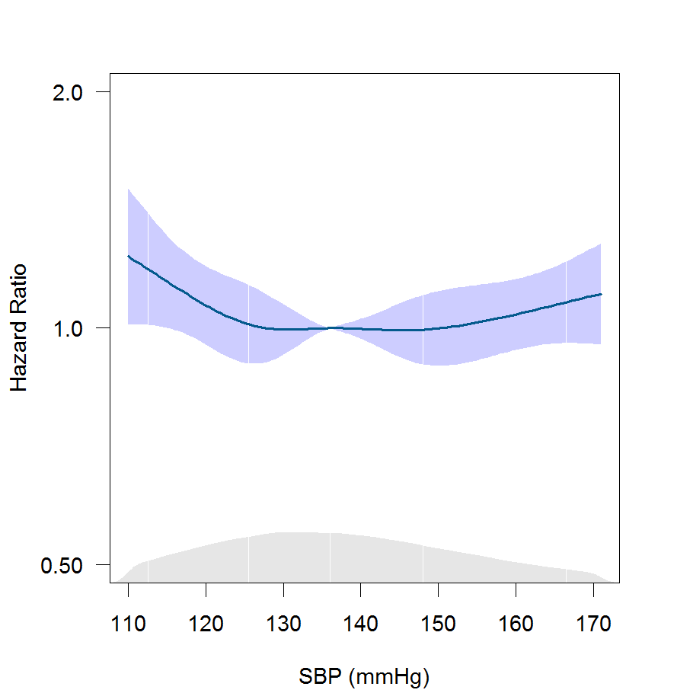 |

BMI, body mass index, SBP, systolic blood pressure

Splines were adjusted for baseline age, ethnicity, smoking status, systolic blood pressure, body mass index, diabetes, Townsend index, lipids lowering drugs, blood pressure lowering drugs. Extreme values in the upper and lower 5% of the lipid trait distribution were excluded

**Supplementary Figure 2: Correlation matrix for lipid traits, using Pearson’s Correlation**


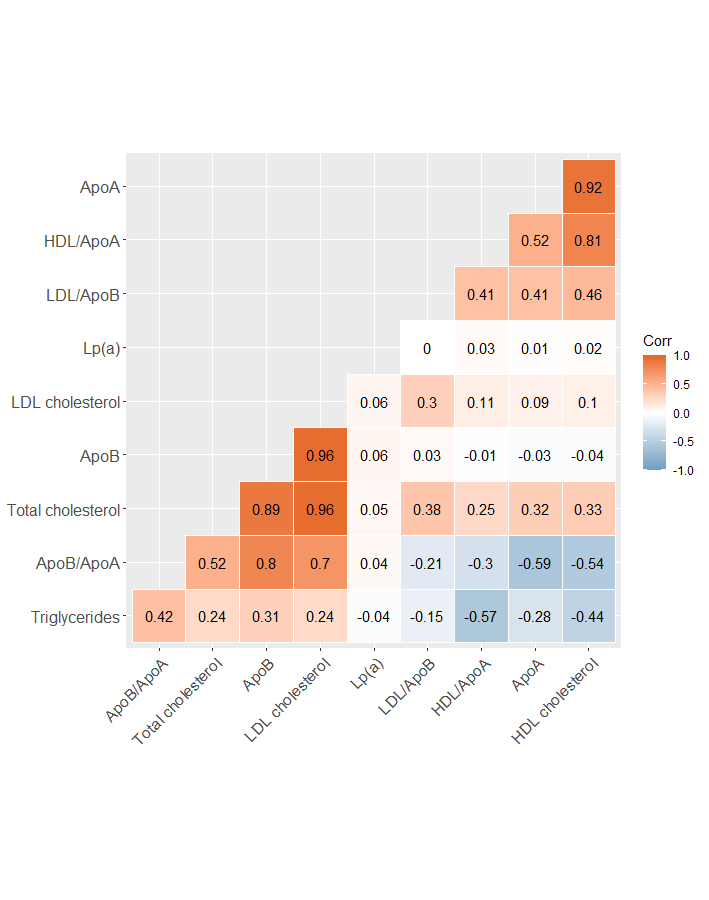


ApoA, apolipoprotein A; ApoB, apolipoprotein B; HDL, high density lipoprotein; LDL, low density lipoprotein; Lp(a), lipoprotein A

**Supplementary Figure 3: Directed acyclic Graph illustrating the causal effect of lipids metabolism on dementia**


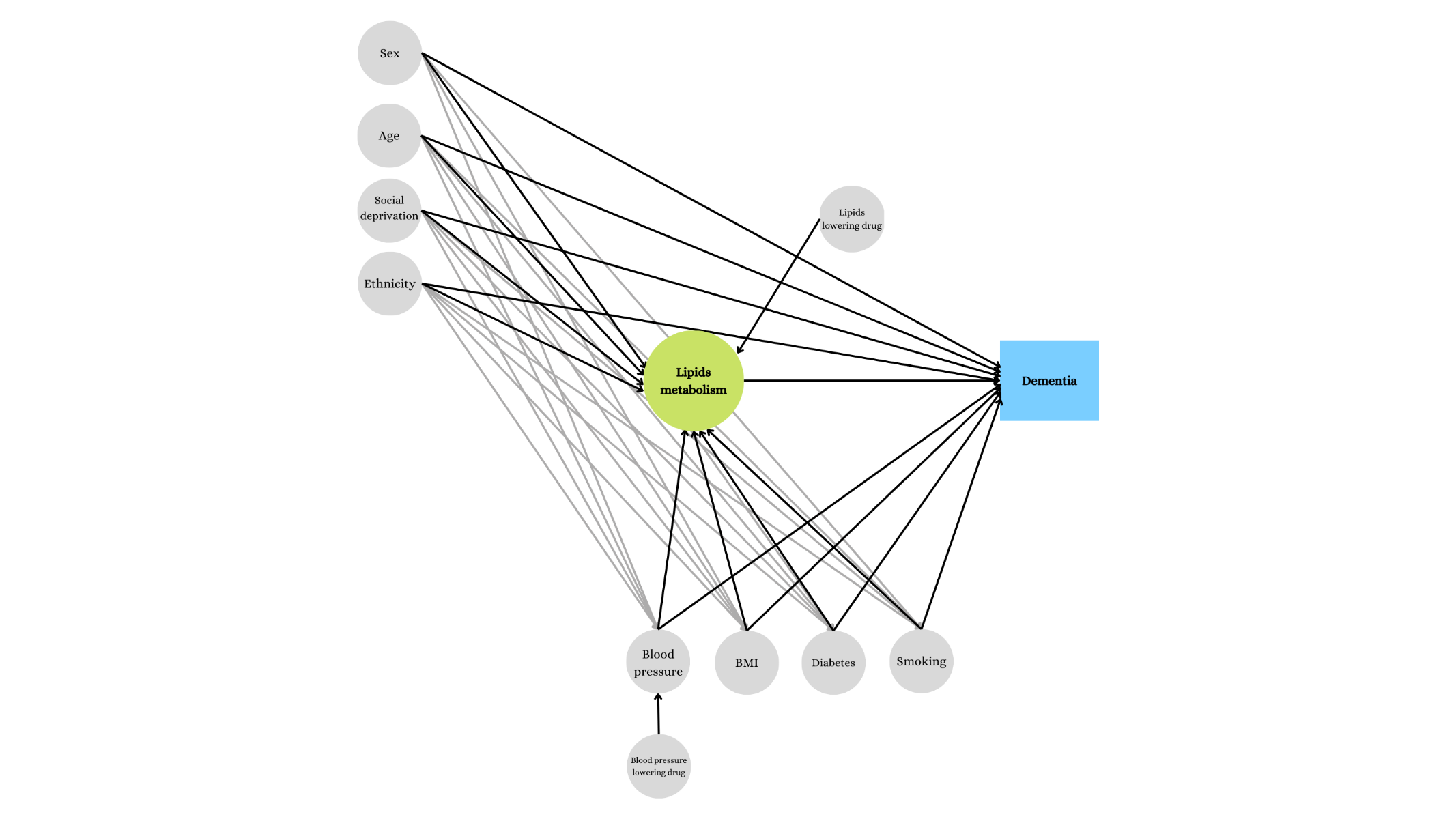


BMI, body mass index

The black arrows indicate the pathways of concern in the study; grey arrows indicate other possible pathways

**Supplementary Figure 4: Multiple-adjusted incident all-cause dementia hazard ratios for lipid traits by quarters, by sex**


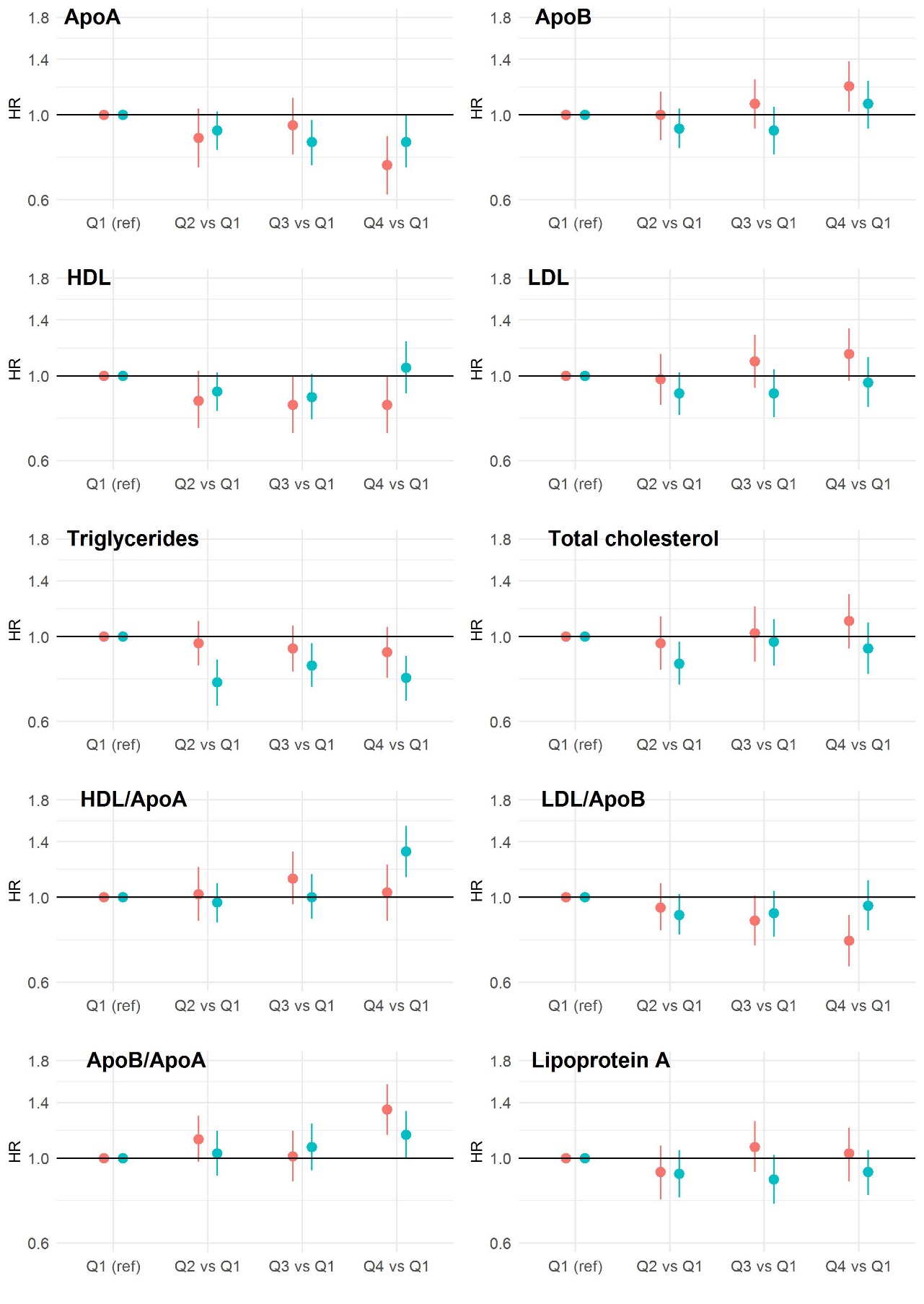


ApoA, apolipoprotein A; ApoB, apolipoprotein B; HDL, high density lipoprotein; LDL, low density lipoprotein; HR, hazard ratio; SD, standard deviation

Adjusted for age, ethnicity, Townsend Deprivation Index, body mass index, systolic blood pressure, smoking status, diabetes, lipids lowering drugs, blood pressure lowering drugs

Pink represents women, and blue represents men

**Supplementary Figure 5: Multiple-adjusted hazard ratios for lipid traits by quarters and standard deviation in associations with the risk of incident all-cause dementia, by age group**


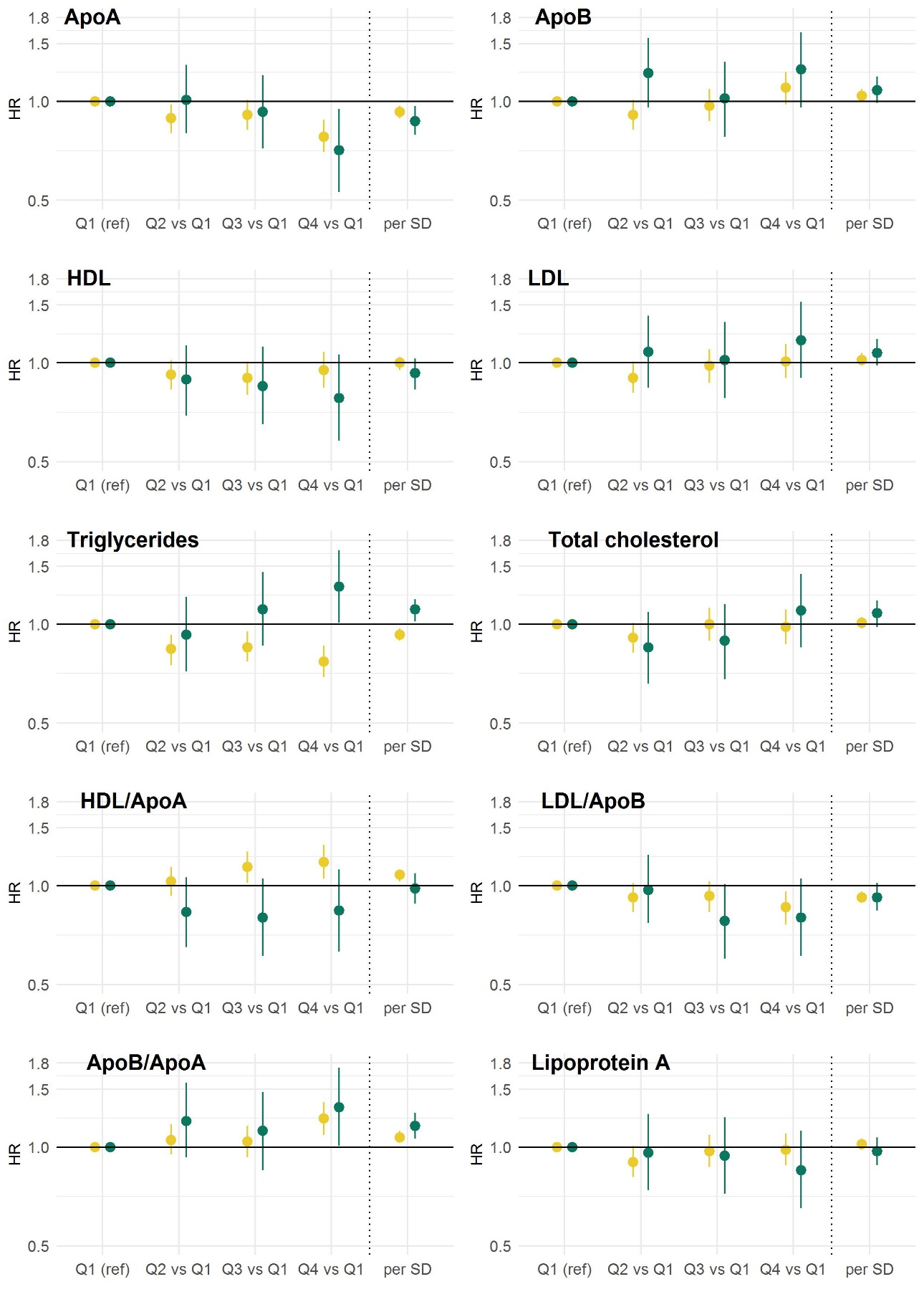


ApoA, apolipoprotein A; ApoB, apolipoprotein B; HDL, high density lipoprotein; LDL, low density lipoprotein; HR, hazard ratio; SD, standard deviation

Adjusted for sex, age, ethnicity, Townsend Deprivation Index, body mass index, systolic blood pressure, smoking status, diabetes, lipids lowering drugs, blood pressure lowering drugs

Yellow represents age ≥60, and green represents age <60

**Supplementary Figure 6: Multiple-adjusted hazard ratios for lipid traits by quarters and standard deviation in associations with the risk of incident all-cause dementia, by socioeconomic status**


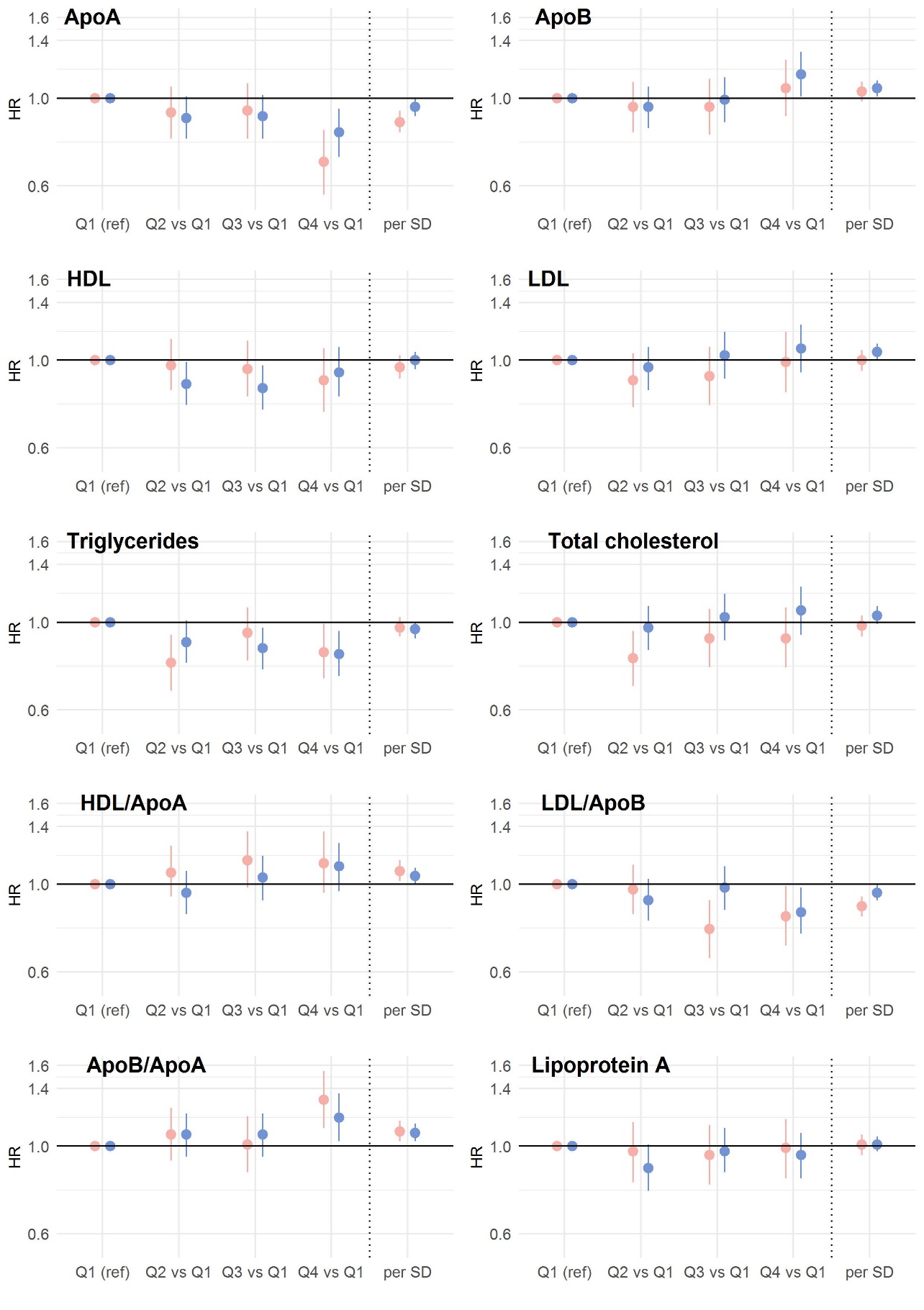


ApoA, apolipoprotein A; ApoB, apolipoprotein B; HDL, high density lipoprotein; LDL, low density lipoprotein; HR, hazard ratio; SD, standard deviation

Adjusted for sex, age, ethnicity, Townsend Deprivation Index, body mass index, systolic blood pressure, smoking status, diabetes, lipids lowering drugs, blood pressure lowering drugs

Purple represents lower socioeconomic status, and pink represents higher socioeconomic status

**Supplementary Table 1: Baseline lipid traits by quarters and number of participants in the UK Biobank**

| **Lipid traits** |  | **Q1** | **Q2** | **Q3** | **Q4** | **N Missing (%)** |
| --- | --- | --- | --- | --- | --- | --- |
| ApoA (g/L) | min, max | 0·42, 1·35 | 1·35, 1,51 | 1·51, 1·70 | 1·70, 2·50 | 42 211 (9·0) |
|  | N | 107 192 | 106 445 | 106 973 | 106 645 |  |
| ApoB (g/L) | min, max | 0·40, 0·86 | 0·86, 1·02 | 1·02, 1·18 | 1·18, 2·00 | 2 536 (5·4) |
|  | N | 116 770 | 117 070 | 116 747 | 116 343 |  |
| HDL cholesterol (mmol/L) | min, max | 0·22, 1·17 | 1·17, 1·40 | 1·40, 1·67 | 1·67, 4·40 | 39 850 (8·5) |
|  | N | 107 524 | 107 580 | 107 332 | 107 179 |  |
| LDL cholesterol (mmol/L) | min, max | 0·27, 2·94 | 2·94, 3·52 | 3·52, 4·12 | 4·12, 9·80 | 1 038 (2·2) |
|  | N | 117 215 | 117 008 | 117 209 | 116 995 |  |
| Triglycerides (mmol/L) | min, max | 0·23, 1·05 | 1·05, 1·48 | 1·48, 2·15 | 2·15, 11·3 | 530 (0·1) |
|  | N | 117 488 | 117 100 | 117 133 | 117 214 |  |
| Total cholesterol (mmol/L) | min, max | 0·60, 4·91 | 4·91, 5·65 | 5·65, 6·42 | 6·42, 15·5 | 156 (0·03) |
|  | N | 117 463 | 117 280 | 117 298 | 117 269 |  |
| HDL/ApoA ratio | min, max | 0·15, 0·33 | 0·33, 0·36 | 0·36, 0·39 | 0·39, 1·41 | 42 408 (9·0) |
|  | N | 106 764 | 106 766 | 106 763 | 106 765 |  |
| LDL/ApoB ratio | min, max | 0·26, 1·27 | 1·27, 1·34 | 1·34, 1·39 | 1·39, 2·72 | 3 457 (0·7) |
|  | N | 116 502 | 116 502 | 116 503 | 116 502 |  |
| ApoB/ApoA ratio | min, max | 1·69, 5·45 | 5·45, 6·67 | 6·67, 8·12 | 8·12, 37·1 | 44 439 (9·5) |
|  | N | 106 256 | 106 258 | 106 256 | 106 257 |  |
| Lp(a) (nmol/L) | min, max | 3·80, 9·58 | 9·58, 21·1 | 21·1, 61·9 | 61·9, 189 | 94 052 (20·0) |
|  | N | 92 507 | 94 090 | 93 614 | 93 852 |  |

ApoA, apolipoprotein A; ApoB, apolipoprotein B; HDL, high density lipoprotein; LDL, low density lipoprotein; Lp(a), lipoprotein A

**Supplementary Table 2: Absolute rate (per 10,000 person-year) of dementia by inpatient diagnosis and diagnosis at death**

|  | **Rates/10,000 person-year (95% CI)** | |
| --- | --- | --- |
|  | **Women** | **Men** |
| Overall n | 1,716 | 2,018 |
| Overall rate | 5·90 (5·53, 6·27) | 8·47 (7·98, 8·96) |
| Inpatient diagnosis n | 1,445 | 1,675 |
| Inpatient diagnosis rate | 4·97 (4·63, 5·31) | 7·02 (6·58, 7·47) |
| Diagnosis at death only* n | 271 | 343 |
| Diagnosis at death only* rate | 0·93 (0·79, 1·08) | 1·45 (1·24, 1·65) |

CI, confidence interval

Sex specific age-adjusted rates of dementia

*Labelled as ‘diagnosis at death only’ if the date of death was equal to the date of dementia

| **Lipid traits** | **Q1 (ref)**  **HR (95% CI)** | **Q2 vs Q1**  **HR (95% CI)** | **Q3 vs Q1**  **HR (95% CI)** | **Q4 vs Q1**  **HR (95% CI)** | **P for trend** | **P (non-linear)** | **per SD**  **HR (95% CI)** |
| --- | --- | --- | --- | --- | --- | --- | --- |
| ApoA | **-** | 0·90 (0·82, 0·99) | 0·91 (0·82, 1·01) | 0·77 (0·69, 0·86) | <0·001 | 0·149 | 0·92 (0·88, 0·95) |
| ApoB | **-** | 0·95 (0·87, 1·05) | 0·98 (0·88, 1·08) | 1·12 (1·01, 1·24) | 0·043 | 0·026 | 1·05 (1·01, 1·09) |
| HDL cholesterol | **-** | 0·91 (0·83, 1·00) | 0·89 (0·81, 0·99) | 0·93 (0·83, 1·04) | 0·119 | 0·189 | 0·99 (0·95, 1·03) |
| LDL cholesterol | **-** | 0·93 (0·84, 1·03) | 0·99 (0·89, 1·10) | 1·04 (0·93, 1·16) | 0·368 | 0·127 | 1·03 (0·99, 1·07) |
| Triglycerides | **-** | 0·86 (0·78, 0·95) | 0·89 (0·81, 0·98) | 0·84 (0·76, 0·93) | 0·003 | 0·055 | 0·96 (0·93, 1·00) |
| Total cholesterol | **-** | 0·90 (0·82, 0·99) | 0·98 (0·88, 1·09) | 1·01 (0·90, 1·12) | 0·651 | 0·046 | 1·02 (0·98, 1·06) |
| HDL/ApoA | **-** | 1·00 (0·91, 1·10) | 1·08 (0·98, 1·20) | 1·12 (1·00, 1·25) | 0·024 | 0·666 | 1·06 (1·02, 1·10) |
| LDL/ApoB | **-** | 0·93 (0·85, 1·02) | 0·90 (0·82, 1·00) | 0·85 (0·76, 0·94) | 0·002 | 0·877 | 0·92 (0·89, 0·96) |
| ApoB/ApoA | **-** | 1·07 (0·97, 1·18) | 1·05 (0·95, 1·16) | 1·23 (1·11, 1·37) | 0·001 | 0·094 | 1·09 (1·05, 1·13) |
| Lp(a) | **-** | 0·91 (0·82, 1·02) | 0·97 (0·87, 1·07) | 0·96 (0·87, 1·07) | 0·725 | 0·256 | 1·01 (0·98, 1·05) |

**Supplementary Table 3: Multiple-adjusted hazard ratios for lipid traits by quarters and per standard deviation, in associations with the risk of incident all-cause dementia**

ApoA, apolipoprotein A; ApoB, apolipoprotein B; HDL, high density lipoprotein; LDL, low density lipoprotein; Lp(a), lipoprotein A; HR, hazard ratio; CI, confidence interval; SD, standard deviation

Adjusted for sex, age, ethnicity, Townsend Deprivation Index, body mass index, systolic blood pressure, smoking status, diabetes, lipids lowering drugs, blood pressure lowering drugs

| **Lipid traits** | **HR (95% CI)** |
| --- | --- |
| ApoA (per 1 g/L) | 0·73 (0·62, 0·84) |
| ApoB (per 1 g/L) | 1·22 (1·05, 1·43) |
| HDL cholesterol (per 1 mmol/L) | 0·96 (0·87, 1·07) |
| LDL cholesterol (per 1 mmol/L) | 1·03 (0·99, 1·08) |
| Triglycerides (per 1 mmol/L) | 0·96 (0·93, 0·99) |
| Total cholesterol (per 1 mmol/L) | 1·02 (0·98, 1·05) |
| HDL/ApoA (per 0·1) | 1·15 (1·05, 1·27) |
| LDL/ApoB (per 0·1) | 0·92 (0·89, 0·96) |
| ApoB/ApoA (per 0·1) | 1·04 (1·02, 1·06) |
| Lp(a) (per 20 nmol/L) | 1·00 (0·99, 1·02) |

**Supplementary Table 4: Multiple-adjusted hazard ratios for lipid traits per unit, in associations with the risk of incident all-cause dementia**

ApoA, apolipoprotein A; ApoB, apolipoprotein B; HDL, high density lipoprotein; LDL, low density lipoprotein; Lp(a), lipoprotein A; HR, hazard ratio; SD, standard deviation; CI, confidence interval

Adjusted for sex, age, ethnicity, Townsend Deprivation Index, body mass index, systolic blood pressure, smoking status, diabetes, lipids lowering drugs, blood pressure lowering drugs

**Supplementary Table 5: Multiple-adjusted hazard ratios for lipid traits by quarters in associations with the risk of incident all-cause dementia, by sex**

| **Lipid traits** | **Subgroup** | **Q1 (ref)**  **HR (95% CI)** | **Q2 vs Q1**  **HR (95% CI)** | **Q3 vs Q1**  **HR (95% CI)** | **Q4 vs Q1**  **HR (95% CI)** | **P for trend** | **P (non-linear)** | **P for interaction** |
| --- | --- | --- | --- | --- | --- | --- | --- | --- |
| ApoA | Women | **-** | 0·87 (0·73, 1·04) | 0·94 (0·79, 1·11) | 0·74 (0·62, 0·88) | <0·001 | 0·025 | 0·702 |
|  | Men | **-** | 0·91 (0·81, 1·02) | 0·85 (0·74, 0·97) | 0·85 (0·73, 1·00) | 0·008 | 0·650 |  |
| ApoB | Women | **-** | 1·00 (0·86, 1·15) | 1·07 (0·92, 1·24) | 1·19 (1·02, 1·38) | 0·014 | 0·537 | 0·709 |
|  | Men | **-** | 0·92 (0·82, 1·04) | 0·91 (0·79, 1·05) | 1·07 (0·92, 1·23) | 0·595 | 0·046 |  |
| HDL cholesterol | Women | **-** | 0·86 (0·73, 1·03) | 0·84 (0·71, 1·00) | 0·84 (0·71, 1·00) | 0·095 | 0·415 | 0·485 |
|  | Men | **-** | 0·91 (0·81, 1·02) | 0·88 (0·77, 1·01) | 1·05 (0·90, 1·23) | 0·651 | 0·032 |  |
| LDL cholesterol | Women | **-** | 0·98 (0·84, 1·14) | 1·09 (0·93, 1·28) | 1·14 (0·97, 1·33) | 0·045 | 0·588 | 0·530 |
|  | Men | - | 0·90 (0·79, 1·02) | 0·90 (0·78, 1·04) | 0·96 (0·83, 1·12) | 0·554 | 0·188 |  |
| Triglycerides | Women | - | 0·96 (0·84, 1·10) | 0·93 (0·81, 1·07) | 0·91 (0·78, 1·06) | 0·184 | 0·988 | 0·474 |
|  | Men | - | 0·76 (0·66, 0·87) | 0·84 (0·74, 0·96) | 0·78 (0·68, 0·89) | 0·005 | 0·003 |  |
| Total cholesterol | Women | - | 0·96 (0·82, 1·13) | 1·02 (0·86, 1·20) | 1·10 (0·93, 1·29) | 0·136 | 0·530 | 0·606 |
|  | Men | - | 0·85 (0·75, 0·97) | 0·97 (0·84, 1·11) | 0·93 (0·80, 1·09) | 0·569 | 0·042 |  |
| HDL/ApoA | Women | - | 1·02 (0·87, 1·20) | 1·12 (0·96, 1·32) | 1·03 (0·87, 1·22) | 0·577 | 0·281 | 0·524 |
|  | Men | - | 0·97 (0·86, 1·09) | 1·00 (0·88, 1·15) | 1·32 (1·13, 1·54) | 0·007 | 0·012 |  |
| LDL/ApoB | Women | - | 0·94 (0·82, 1·09) | 0·87 (0·75, 1·01) | 0·77 (0·66, 0·90) | <0·001 | 0·809 | 0·206 |
|  | Men | - | 0·90 (0·80, 1·02) | 0·91 (0·79, 1·04) | 0·95 (0·82, 1·11) | 0·361 | 0·257 |  |
| ApoB/ApoA | Women | - | 1·12 (0·98, 1·29) | 1·01 (0·87, 1·18) | 1·34 (1·15, 1·56) | 0·003 | 0·016 | 0·685 |
|  | Men | - | 1·03 (0·90, 1·18) | 1·07 (0·93, 1·23) | 1·15 (1·00, 1·33) | 0·051 | 0·873 |  |
| Lp(a) | Women | **-** | 0·92 (0·78, 1·08) | 1·07 (0·92, 1·25) | 1·03 (0·87, 1·20) | 0·355 | 0·222 | 0·089 |
|  | Men | **-** | 0·91 (0·79, 1·05) | 0·88 (0·76, 1·02) | 0·92 (0·80, 1·05) | 0·184 | 0·450 |  |

ApoA, apolipoprotein A; ApoB, apolipoprotein B; HDL, high density lipoprotein; LDL, low density lipoprotein; Lp(a), lipoprotein A; SD, standard deviation; HR, hazard ratio; CI, confidence interval

Adjusted for age, ethnicity, Townsend Deprivation Index, body mass index, systolic blood pressure, smoking status, diabetes, lipids lowering drugs, blood pressure lowering drugs

**Supplementary Table 6: Multiple-adjusted hazard ratios for lipid traits by quarters and standard deviation in associations with the risk of incident all-cause dementia, by age group**

| **Lipid traits** | **Subgroup** | **Q1 (ref)**  **HR (95% CI)** | **Q2 vs Q1**  **HR (95% CI)** | **Q3 vs Q1**  **HR (95% CI)** | **Q4 vs Q1**  **HR (95% CI)** | **P for trend** | **P (non-linear)** | **P for interaction** | **per SD**  **HR (95% CI)** |
| --- | --- | --- | --- | --- | --- | --- | --- | --- | --- |
| ApoA | ≥60 years | **-** | 0·89 (0·80, 0·98) | 0·91 (0·82, 1·01) | 0·78 (0·70, 0·88) | <0·001 | 0·164 | 0·506 | 0·93 (0·89, 0·97) |
|  | <60 years | **-** | 1·01 (0·80, 1·29) | 0·93 (0·72, 1·20) | 0·71 (0·53, 0·95) | 0·024 | 0·315 |  | 0·87 (0·79, 0·97) |
| ApoB | ≥60 years | **-** | 0·91 (0·82, 1·01) | 0·97 (0·87, 1·09) | 1·10 (0·98, 1·23) | 0·078 | 0·012 | 0·770 | 1·04 (1·00, 1·09) |
|  | <60 years | **-** | 1·22 (0·96, 1·56) | 1·02 (0·78, 1·32) | 1·25 (0·96, 1·62) | 0·267 | 0·139 |  | 1·08 (0·99, 1·19) |
| HDL cholesterol | ≥60 years | **-** | 0·92 (0·83, 1·02) | 0·90 (0·80, 1·01) | 0·95 (0·84, 1·08) | 0·353 | 0·080 | 0·267 | 1·00 (0·95, 1·04) |
|  | <60 years | **-** | 0·89 (0·69, 1·13) | 0·85 (0·65, 1·12) | 0·78 (0·58, 1·06) | 0·105 | 0·923 |  | 0·93 (0·83, 1·03) |
| LDL cholesterol | ≥60 years | **-** | 0·90 (0·81, 1·01) | 0·98 (0·87, 1·10) | 1·01 (0·90, 1·14) | 0·559 | 0·080 | 0·511 | 1·02 (0·98, 1·07) |
|  | <60 years | **-** | 1·08 (0·84, 1·39) | 1·02 (0·78, 1·33) | 1·17 (0·90, 1·53) | 0·319 | 0·668 |  | 1·07 (0·98, 1·18) |
| Triglycerides | ≥60 years | **-** | 0·84 (0·75, 0·93) | 0·85 (0·77, 0·95) | 0·77 (0·69, 0·86) | <0·001 | 0·088 | <0·001 | 0·93 (0·89, 0·97) |
|  | <60 years | **-** | 0·93 (0·72, 1·21) | 1·11 (0·86, 1·44) | 1·30 (1·01, 1·68) | 0·016 | 0·358 |  | 1·11 (1·02, 1·19) |
| Total cholesterol | ≥60 years | **-** | 0·91 (0·82, 1·01) | 1·00 (0·89, 1·12) | 0·98 (0·87, 1·11) | 0·880 | 0·120 | 0·438 | 1·01 (0·97, 1·05) |
|  | <60 years | **-** | 0·85 (0·66, 1·09) | 0·89 (0·68, 1·15) | 1·10 (0·85, 1·42) | 0·359 | 0·103 |  | 1·08 (0·98, 1·18) |
| HDL/ApoA | ≥60 years | **-** | 1·03 (0·93, 1·14) | 1·14 (1·02, 1·27) | 1·18 (1·05, 1·33) | 0·003 | 0·770 | 0·022 | 1·08 (1·03, 1·12) |
|  | <60 years | **-** | 0·83 (0·65, 1·06) | 0·80 (0·61, 1·05) | 0·84 (0·63, 1·12) | 0·206 | 0·431 |  | 0·98 (0·88, 1·09) |
| LDL/ApoB | ≥60 years | **-** | 0·92 (0·83, 1·02) | 0·93 (0·83, 1·03) | 0·86 (0·76, 0·96) | 0·014 | 0·603 | 0·369 | 0·92 (0·89, 0·96) |
|  | <60 years | **-** | 0·92 (0·83, 1·02) | 0·93 (0·83, 1·03) | 0·86 (0·76, 0·96) | 0·041 | 0·516 |  | 0·92 (0·84, 1·02) |
| ApoB/ApoA | ≥60 years | **-** | 1·05 (0·95, 1·17) | 1·04 (0·93, 1·16) | 1·22 (1·09, 1·37) | 0·002 | 0·138 | 0·697 | 1·07 (1·03, 1·12) |
|  | <60 years | **-** | 1·20 (0·93, 1·57) | 1·12 (0·85, 1·47) | 1·32 (1·01, 1·74) | 0·082 | 0·476 |  | 1·16 (1·06, 1·27) |
| Lp(a) | ≥60 years | **-** | 0·90 (0·81, 1·01) | 0·97 (0·87, 1·09) | 0·98 (0·88, 1·10) | 0·939 | 0·188 | 0·283 | 1·02 (0·98, 1·06) |
|  | <60 years | **-** | 0·96 (0·74, 1·26) | 0·94 (0·72, 1·23) | 0·85 (0·65, 1·12) | 0·259 | 0·936 |  | 0·97 (0·88, 1·07) |

ApoA, apolipoprotein A; ApoB, apolipoprotein B; HDL, high density lipoprotein; LDL, low density lipoprotein; Lp(a), lipoprotein A; SD, standard deviation; HR, hazard ratio; CI, confidence interval

Adjusted for sex, age, ethnicity, Townsend Deprivation Index, body mass index, systolic blood pressure, smoking status, diabetes, lipids lowering drugs, blood pressure lowering drugs

**Supplementary Table 7: Multiple-adjusted hazard ratios for lipid traits by quarters and standard deviation in associations with the risk of incident all-cause dementia, by socioeconomic status**

| **Lipid traits** | **Subgroup** | **Q1 (ref)**  **HR (95% CI)** | **Q2 vs Q1**  **HR (95% CI)** | **Q3 vs Q1**  **HR (95% CI)** | **Q4 vs Q1**  **HR (95% CI)** | **P for trend** | **P (non-linear)** | **P for interaction** | **per SD**  **HR (95% CI)** |
| --- | --- | --- | --- | --- | --- | --- | --- | --- | --- |
| ApoA | Higher SES | **-** | 0·89 (0·79, 1·01) | 0·90 (0·79, 1·02) | 0·82 (0·71, 0·94) | <0·001 | 0·524 | 0·236 | 0·95 (0·90, 1·00) |
|  | Lower SES | **-** | 0·92 (0·79, 1·07) | 0·93 (0·79, 1·09) | 0·69 (0·57, 0·83) | 0·010 | 0·070 |  | 0·87 (0·82, 0·93) |
| ApoB | Higher SES | **-** | 0·95 (0·84, 1·07) | 0·99 (0·87, 1·13) | 1·15 (1·01, 1·31) | 0·590 | 0·073 | 0·367 | 1·06 (1·01, 1·11) |
|  | Lower SES | **-** | 0·95 (0·82, 1·10) | 0·95 (0·81, 1·12) | 1·06 (0·90, 1·25) | 0·033 | 0·364 |  | 1·04 (0·98, 1·10) |
| HDL cholesterol | Higher SES | **-** | 0·87 (0·77, 0·99) | 0·85 (0·75, 0·97) | 0·93 (0·81, 1·08) | 0·242 | 0·040 | 0·796 | 1·00 (0·95, 1·05) |
|  | Lower SES | **-** | 0·97 (0·84, 1·13) | 0·95 (0·81, 1·12) | 0·89 (0·74, 1·07) | 0·302 | 0·930 |  | 0·96 (0·90, 1·03) |
| LDL cholesterol | Higher SES | **-** | 0·96 (0·84, 1·08) | 1·03 (0·90, 1·18) | 1·07 (0·93, 1·23) | 0·910 | 0·474 | 0·392 | 1·05 (1·00, 1·10) |
|  | Lower SES | **-** | 0·89 (0·76, 1·04) | 0·91 (0·77, 1·08) | 0·99 (0·83, 1·18) | 0·219 | 0·217 |  | 1·00 (0·94, 1·06) |
| Triglycerides | Higher SES | **-** | 0·89 (0·79, 1·01) | 0·86 (0·76, 0·97) | 0·83 (0·73, 0·95) | 0·230 | 0·599 | 0·428 | 0·96 (0·91, 1·00) |
|  | Lower SES | **-** | 0·79 (0·67, 0·93) | 0·94 (0·80, 1·09) | 0·84 (0·72, 0·99) | 0·005 | 0·014 |  | 0·97 (0·92, 1·03) |
| Total cholesterol | Higher SES | **-** | 0·97 (0·85, 1·10) | 1·03 (0·90, 1·18) | 1·07 (0·93, 1·23) | 0·466 | 0·597 | 0·194 | 1·04 (0·99, 1·10) |
|  | Lower SES | **-** | 0·81 (0·69, 0·95) | 0·91 (0·77, 1·08) | 0·91 (0·77, 1·09) | 0·243 | 0·034 |  | 0·98 (0·92, 1·04) |
| HDL/ApoA | Higher SES | **-** | 0·95 (0·84, 1·08) | 1·04 (0·91, 1·18) | 1·11 (0·96, 1·27) | 0·104 | 0·336 | 0·784 | 1·05 (1·00, 1·10) |
|  | Lower SES | **-** | 1·07 (0·93, 1·25) | 1·15 (0·98, 1·36) | 1·13 (0·95, 1·36) | 0·107 | 0·723 |  | 1·08 (1·02, 1·15) |
| LDL/ApoB | Higher SES | **-** | 0·91 (0·81, 1·03) | 0·98 (0·86, 1·11) | 0·85 (0·75, 0·98) | 0·006 | 0·152 | 0·266 | 0·95 (0·91, 1·00) |
|  | Lower SES | **-** | 0·97 (0·84, 1·12) | 0·77 (0·65, 0·91) | 0·83 (0·70, 0·99) | 0·073 | 0·147 |  | 0·88 (0·83, 0·93) |
| ApoB/ApoA | Higher SES | **-** | 1·07 (0·94, 1·21) | 1·07 (0·94, 1·21) | 1·18 (1·03, 1·36) | 0·007 | 0·654 | 0·493 | 1·08 (1·03, 1·14) |
|  | Lower SES | **-** | 1·07 (0·92, 1·25) | 1·01 (0·86, 1·19) | 1·31 (1·11, 1·55) | 0·026 | 0·061 |  | 1·09 (1·03, 1·16) |
| Lp(a) | Higher SES | **-** | 0·88 (0·77, 1·01) | 0·97 (0·86, 1·11) | 0·95 (0·83, 1·08) | 0·838 | 0·168 | 0·976 | 1·01 (0·97, 1·06) |
|  | Lower SES | **-** | 0·97 (0·81, 1·15) | 0·95 (0·80, 1·13) | 0·99 (0·83, 1·17) | 0·757 | 0·848 |  | 1·01 (0·95, 1·07) |

ApoA, apolipoprotein A; ApoB, apolipoprotein B; HDL, high density lipoprotein; LDL, low density lipoprotein; Lp(a), lipoprotein A; SD, standard deviation; HR, hazard ratio; CI, confidence interval; SES, socioeconomic status

Adjusted for sex, age, ethnicity, Townsend Deprivation Index, body mass index, systolic blood pressure, smoking status, diabetes, lipids lowering drugs, blood pressure lowering drugs

**Supplementary Table 8: Multiple-adjusted hazard ratios for lipid traits by quarters and per standard deviation, in associations with the risk of incident vascular dementia and Alzheimer’s disease**

| **Lipid traits** | **Dementia subtypes** | **Q1 (ref)**  **HR (95% CI)** | **Q2 vs Q1**  **HR (95% CI)** | **Q3 vs Q1**  **HR (95% CI)** | **Q4 vs Q1**  **HR (95% CI)** | **P for trend** | **P (non-linear)** | **per SD**  **HR (95% CI)** |
| --- | --- | --- | --- | --- | --- | --- | --- | --- |
| ApoA | Vascular dementia | **-** | 0·88 (0·73, 1·06) | 0·89 (0·73, 1·09) | 0·69 (0·55, 0·86) | 0·003 | 0·268 | 0·91 (0·84, 0·99) |
|  | Alzheimer’s disease | **-** | 1·05 (0·88, 1·24) | 0·90 (0·75, 1·08) | 0·82 (0·68, 1·00) | 0·019 | 0·332 | 0·92 (0·86, 0·99) |
| ApoB | Vascular dementia | **-** | 0·97 (0·81, 1·17) | 0·93 (0·75, 1·15) | 1·42 (1·15, 1·74) | 0·003 | 0·002 | 1·11 (1·03, 1·20) |
|  | Alzheimer’s disease | **-** | 1·06 (0·90, 1·25) | 1·06 (0·89, 1·27) | 1·32 (1·11, 1·59) | 0·003 | 0·232 | 1·11 (1·04, 1·18) |
| HDL cholesterol | Vascular dementia | **-** | 0·84 (0·69, 1·02) | 0·90 (0·73, 1·10) | 0·94 (0·76, 1·18) | 0·655 | 0·186 | 1·01 (0·93, 1·09) |
|  | Alzheimer’s disease | **-** | 0·97 (0·82, 1·15) | 0·86 (0·72, 1·03) | 0·90 (0·74, 1·10) | 0·182 | 0·514 | 0·96 (0·90, 1·04) |
| LDL cholesterol | Vascular dementia | **-** | 0·99 (0·82, 1·20) | 0·95 (0·76, 1·19) | 1·37 (1·10, 1·71) | 0·009 | 0·018 | 1·10 (1·02, 1·19) |
|  | Alzheimer’s disease | **-** | 0·89 (0·75, 1·06) | 1·03 (0·86, 1·24) | 1·13 (0·94, 1·36) | 0·086 | 0·124 | 1·07 (1·00, 1·14) |
| Triglycerides | Vascular dementia | **-** | 0·99 (0·82, 1·21) | 0·91 (0·75, 1·11) | 0·91 (0·75, 1·12) | 0·265 | 0·833 | 0·97 (0·90, 1·04) |
|  | Alzheimer’s disease | **-** | 0·95 (0·80, 1·11) | 0·94 (0·80, 1·11) | 0·84 (0·70, 1·00) | 0·059 | 0·735 | 0·96 (0·90, 1·02) |
| Total cholesterol | Vascular dementia | **-** | 1·01 (0·84, 1·23) | 1·02 (0·82, 1·27) | 1·29 (1·03, 1·61) | 0·035 | 0·254 | 1·09 (1·01, 1·18) |
|  | Alzheimer’s disease | **-** | 0·96 (0·81, 1·14) | 1·02 (0·85, 1·23) | 1·12 (0·93, 1·35) | 0·167 | 0·514 | 1·05 (0·99, 1·13) |
| HDL/ApoA | Vascular dementia | **-** | 0·92 (0·76, 1·12) | 1·17 (0·96, 1·43) | 1·37 (1·10, 1·70) | 0·002 | 0·106 | 1·16 (1·08, 1·25) |
|  | Alzheimer’s disease | **-** | 0·93 (0·78, 1·10) | 1·12 (0·94, 1·34) | 1·01 (0·83, 1·23) | 0·467 | 0·124 | 1·01 (0·94, 1·08) |
| LDL/ApoB | Vascular dementia | **-** | 0·95 (0·79, 1·14) | 1·01 (0·82, 1·23) | 0·83 (0·67, 1·04) | 0·211 | 0·400 | 0·93 (0·87, 1·01) |
|  | Alzheimer’s disease | **-** | 0·83 (0·71, 0·98) | 0·86 (0·73, 1·02) | 0·66 (0·55, 0·80) | <0·001 | 0·136 | 0·86 (0·80, 0·92) |
| ApoB/ApoA | Vascular dementia | **-** | 1·02 (0·84, 1·24) | 1·07 (0·87, 1·31) | 1·35 (1·10, 1·67) | 0·008 | 0·283 | 1·13 (1·04, 1·22) |
|  | Alzheimer’s disease | **-** | 1·22 (1·03, 1·44) | 1·07 (0·89, 1·28) | 1·37 (1·14, 1·65) | 0·008 | 0·032 | 1·12 (1·05, 1·20) |
| Lp(a) | Vascular dementia | **-** | 0·92 (0·74, 1·13) | 1·06 (0·87, 1·30) | 0·91 (0·73, 1·13) | 0·704 | 0·245 | 0·97 (0·90, 1·05) |
|  | Alzheimer’s disease | **-** | 0·97 (0·81, 1·17) | 1·02 (0·85, 1·22) | 0·90 (0·75, 1·09) | 0·417 | 0·523 | 0·97 (0·91, 1·04) |

ApoA, apolipoprotein A; ApoB, apolipoprotein B; HDL, high density lipoprotein; LDL, low density lipoprotein; Lp(a), lipoprotein A; SD, standard deviation; HR, hazard ratio; CI, confidence interval

Adjusted for sex, age, ethnicity, Townsend Deprivation Index, body mass index, systolic blood pressure, smoking status, diabetes, lipids lowering drugs, blood pressure lowering drug

| **Lipid traits** | **C-statistic** | **Difference in c-statistic from base model + LDL cholesterol (per SD) x 10^-3^** |
| --- | --- | --- |
| Base model* + LDL cholesterol (per SD) | 0·803673 | NA |
| Base model* + ApoA (per SD) | 0·804203 | 0·5299 |
| Base model* + ApoB (per SD) | 0·803742 | 0·0692 |
| Base model* + HDL cholesterol (per SD) | 0·803800 | 0·1272 |
| Base model* + Triglycerides (per SD) | 0·803760 | 0·0871 |
| Base model* + Total cholesterol (per SD) | 0·803649 | -0·0239 |
| Base model* + HDL/ApoA (per SD) | 0·803718 | 0·0448 |
| Base model* + LDL/ApoB (per SD) | 0·803978 | 0·3051 |
| Base model* + ApoB/ApoA (per SD) | 0·804043 | 0·3706 |
| Base model* + Lp(a) (per SD) | 0·803681 | 0·0083 |

**Supplementary Table 9: Prognosis value using c-statistics and differences of lipid trait (per SD) and incident all-cause dementia compared with traditional risk factors plus LDL cholesterol**

ApoA, apolipoprotein A; ApoB, apolipoprotein B; HDL, high density lipoprotein; LDL, low density lipoprotein; Lp(a), lipoprotein A; SD, standard deviation; HR, hazard ratio

*sex, age, ethnicity, Townsend Deprivation Index, body mass index, systolic blood pressure, smoking status, diabetes, lipids lowering drugs, blood pressure lowering drug

**Supplementary Table 10: Prognosis value using c-statistics and differences of lipid trait (per SD) and incident all-cause dementia compared with traditional risk factors plus total cholesterol**

| **Lipid traits** | **C-statistic** | **Difference in c-statistic from base model + Total cholesterol (per SD) x 10^-3^** |
| --- | --- | --- |
| Base model* + Total cholesterol (per SD) | 0·803649 | NA |
| Base model* + ApoA (per SD) | 0·804203 | 0·5538 |
| Base model* + ApoB (per SD) | 0·803742 | 0·0931 |
| Base model* + HDL cholesterol (per SD) | 0·803800 | 0·1511 |
| Base model* + LDL cholesterol (per SD) | 0·803673 | 0·0239 |
| Base model* + Triglycerides (per SD) | 0·803760 | 0·1109 |
| Base model* + HDL/ApoA (per SD) | 0·803718 | 0·0687 |
| Base model* + LDL/ApoB (per SD) | 0·803978 | 0·3290 |
| Base model* + ApoB/ApoA (per SD) | 0·804043 | 0·3945 |
| Base model* + Lp(a) (per SD) | 0·803681 | 0·0322 |

ApoA, apolipoprotein A; ApoB, apolipoprotein B; HDL, high density lipoprotein; LDL, low density lipoprotein; Lp(a), lipoprotein A; SD, standard deviation; HR, hazard ratio

*sex, age, ethnicity, Townsend Deprivation Index, body mass index, systolic blood pressure, smoking status, diabetes, lipids lowering drugs, blood pressure lowering drugs

**Supplementary Table 11: Multiple-adjusted hazard ratios for lipid traits by quarters, in associations with the risk of incident all-cause dementia, after excluding five years***

| **Lipid traits** | **Q1 (ref)**  **HR (95% CI)** | **Q2 vs Q1**  **HR (95% CI)** | **Q3 vs Q1**  **HR (95% CI)** | **Q4 vs Q1**  **HR (95% CI)** | **per SD**  **HR (95% CI)** |
| --- | --- | --- | --- | --- | --- |
| ApoA | - | 0·89 (0·80, 0·99) | 0·90 (0·81, 1·01) | 0·72 (0·64, 0·82) | 0·90 (0·86, 0·94) |
| ApoB | - | 0·96 (0·87, 1·07) | 0·99 (0·89, 1·11) | 1·17 (1·04, 1·31) | 1·06 (1·02, 1·11) |
| HDL cholesterol | - | 0·88 (0·79, 0·98) | 0·88 (0·78, 0·98) | 0·85 (0·75, 0·97) | 0·95 (0·91, 1·00) |
| LDL cholesterol | - | 0·92 (0·83, 1·03) | 0·98 (0·87, 1·11) | 1·04 (0·92, 1·18) | 1·03 (0·99, 1·08) |
| Triglycerides | - | 0·83 (0·75, 0·93) | 0·87 (0·78, 0·97) | 0·87 (0·78, 0·97) | 0·98 (0·95, 1·03) |
| Total cholesterol | - | 0·91 (0·81, 1·01) | 0·97 (0·86, 1·09) | 1·01 (0·89, 1·14) | 1·02 (0·97, 1·06) |
| HDL/ApoA | - | 0·97 (0·87, 1·08) | 1·02 (0·91, 1·14) | 1·04 (0·92, 1·18) | 1·03 (0·98, 1·07) |
| LDL/ApoB | - | 0·91 (0·82, 1·01) | 0·89 (0·79, 0·99) | 0·80 (0·71, 0·91) | 0·90 (0·87, 0·94) |
| ApoB/ApoA | - | 1·09 (0·98, 1·21) | 1·10 (0·98, 1·23) | 1·26 (1·12, 1·42) | 1·10 (1·06, 1·15) |
| Lp(a) | - | 0·90 (0·80, 1·02) | 0·98 (0·87, 1·10) | 0·99 (0·88, 1·11) | 1·02 (0·98, 1·06) |

ApoA, apolipoprotein A; ApoB, apolipoprotein B; HDL, high density lipoprotein; LDL, low density lipoprotein; Lp(a), lipoprotein A; SD, standard deviation; HR, hazard ratio; CI, confidence interval

Adjusted for sex, age, ethnicity, Townsend Deprivation Index, body mass index, systolic blood pressure, smoking status, diabetes, lipids lowering drugs, blood pressure lowering drugs

*Dataset comprises 460,897 participants (251,230 women), with 2,972 dementia cases (1,396 women)

**Supplementary Table 12: Multiple-adjusted sub-distribution hazard ratios (sHR) for lipid traits by quarters and standard deviation, in associations with the risk of incident all-cause dementia, using Fine-Gray competing risk models**

| **Lipid traits** | **Q1 (ref)**  **sHR (95% CI)** | **Q2 vs Q1**  **sHR (95% CI)** | **Q3 vs Q1**  **sHR (95% CI)** | **Q4 vs Q1**  **sHR (95% CI)** | **Per SD**  **sHR (95% CI)** |
| --- | --- | --- | --- | --- | --- |
| ApoA | **-** | 0·91 (0·83, 1·00) | 0·92 (0·83, 1·02) | 0·78 (0·70, 0·87) | 0·92 (0·89, 0·96) |
| ApoB | **-** | 0·96 (0·87, 1·05) | 0·98 (0·88, 1·08) | 1·11 (1·00, 1·23) | 1·05 (1·01, 1·09) |
| HDL cholesterol | **-** | 0·91 (0·83, 1·01) | 0·89 (0·81, 0·99) | 0·93 (0·83, 1·04) | 0·99 (0·95, 1·03) |
| LDL cholesterol | **-** | 0·94 (0·85, 1·03) | 0·98 (0·88, 1·10) | 1·03 (0·93, 1·15) | 1·03 (0·99, 1·07) |
| Triglycerides | **-** | 0·85 (0·77, 0·94) | 0·88 (0·80, 0·97) | 0·83 (0·75, 0·92) | 0·96 (0·93, 1·00) |
| Total cholesterol | **-** | 0·90 (0·82, 1·00) | 0·98 (0·88, 1·09) | 1·00 (0·89, 1·12) | 1·02 (0·98, 1·06) |
| HDL/ApoA | **-** | 0·99 (0·90, 1·09) | 1·08 (0·97, 1·19) | 1·12 (1·00, 1·25) | 1·06 (1·02, 1·11) |
| LDL/ApoB | **-** | 0·94 (0·84, 1·04) | 0·91 (0·81, 1·02) | 0·85 (0·75, 0·96) | 0·93 (0·89, 0·97) |
| ApoB/ApoA | **-** | 1·06 (0·96, 1·17) | 1·02 (0·92, 1·13) | 1·19 (1·07, 1·32) | 1·08 (1·04, 1·12) |
| Lp(a) | **-** | 0·91 (0·82, 1·01) | 0·97 (0·87, 1·07) | 0·96 (0·86, 1·06) | 1·01 (0·97, 1·05) |

ApoA, apolipoprotein A; ApoB, apolipoprotein B; HDL, high density lipoprotein; LDL, low density lipoprotein; Lp(a), lipoprotein A; sHR, sub-distribution hazard ratio; CI, confidence interval; SD, standard deviation

Adjusted for sex, age, ethnicity, Townsend Deprivation Index, body mass index, systolic blood pressure, smoking status, diabetes, lipids lowering drugs, blood pressure lowering drugs
